# Dengue epidemiology and transmission intensity across Panama during 2000–2024: a modelling study

**DOI:** 10.1016/j.lana.2026.101471

**Published:** 2026-04-22

**Authors:** Mario Quijada, Anna Vicco, Fulvia Bajura, Lourdes Moreno, Yamilka Díaz, Daniel Laydon, Lizbeth Cerezo, Reina Roa, Ilaria Dorigatti

**Affiliations:** a Department of Infectious Disease Epidemiology, School of Public Health, Faculty of Medicine, Imperial College London, London, United Kingdom; b Estación Biomédica Experimental, Instituto Conmemorativo Gorgas de Estudios de La Salud, Panama; c Ministerio de Salud de Panamá, Panama; d Departamento de Investigación en Virología y Biotecnología, Instituto Conmemorativo Gorgas de Estudios de La Salud, Panama

**Keywords:** Dengue epidemiology, Force of infection, Spatial heterogeneity, Seroprevalence

## Abstract

**Background:**

Panama is a dengue endemic country which experienced a large outbreak in 2024 with over 32,000 reported cases and an incidence rate exceeding 700 cases per 100,000 inhabitants. Despite decades of circulation, the epidemiology of dengue and its heterogeneity in transmission intensity across Panama have not yet been characterised.

**Methods:**

We used 25 years of dengue case notification and population data from across Panama's 16 health regions and 82 districts to characterise dengue epidemiology and transmission intensity in the country. The analytic dataset comprised 128,890 dengue cases, of whom 52% were female and 48% were male; the mean age was 32.4 years (range 0–108 years). Ethnicity data are not collected in Panama's national dengue surveillance system and were therefore unavailable for this analysis. We characterised spatial heterogeneities in delay distributions by fitting parametric probability distributions to epidemiological delays, and demographic differences in the incidence risk ratio of dengue, and of dengue attributable hospitalisations and deaths. We also implemented catalytic models to infer the time-constant dengue force-of-infection (FOI) (i.e. the long-term average annual per capita risk of infection for a susceptible individual) from the age-stratified case notification data reported across Panama during 2000–2024 and explored age- and sex-related differences in dengue case reporting in sensitivity analyses.

**Findings:**

We observed spatial variation in delay distributions across health regions. The mean of the regional average time from symptoms onset to (i) reporting was 4.78 days (95% CI: 4.72–4.84 days), (ii) hospitalisation was 4.49 days (95% CI: 4.22–4.76), and (iii) recovery was 7.82 days (95% CI: 6.47–8.85 days). The dengue transmission intensity also showed spatial heterogeneity, with a mean regional per-serotype FOI of 0.008 (95% CrI: 0.004–0.015). The mean regional probability of detecting a secondary case was 0.415 (95% CrI: 0.233–0.871) and the probability of detecting a primary case relative to a secondary case was 0.131 (95% CrI: 0.049–0.348). We found evidence of age- and sex-related differences in dengue reporting. Given the estimated seroprevalence at nine years (obtained from the analysis of case-notification data) is below 40% across the whole country, dengue vaccination with TAK-003 is currently not recommended in Panama according to the WHO guidelines. In the future, age-stratified seroprevalence surveys would be useful to validate these estimates.

**Interpretation:**

This analysis provides a characterisation of dengue epidemiology and estimates of dengue FOI and infection burden in Panama across the last 25 years. This work will inform policy decisions at the Ministry of Health of Panama, providing guidance on resource allocation to strengthen the local surveillance system and to decision making on the potential implementation of new interventions.

**Funding:**

SENACYT-IFARHU (Panamá), 10.13039/501100000265UK Medical Research Council and 10.13039/100010269Wellcome Trust.

**Research in context**

**Evidence before this study**

To contextualise this study within the published literature, we searched PubMed and Web of Science using the terms (“arbovirus” OR “dengue”) AND (“Panama”) AND (“modelling” OR “mathematical model” OR “statistical model”) AND (“case notification” OR “surveillance data”) without language restrictions, including published papers from 1993 to 2025. We identified one modelling study analysing arbovirus case notification data in Panama. Most published research focused on dengue genomic surveillance and vector control strategies. None of the retrieved studies characterised dengue transmission intensity in the country.

**Added value of this study**

This study reconstructs the epidemiology of dengue virus (DENV) in Panama from 2000 to 2024 using line list data of laboratory-confirmed cases. It provides a comprehensive analysis of dengue incidence, hospitalisation rate, case fatality ratios (CFR), and human delay distributions across Panama's 16 health regions and 82 districts. Furthermore, this work provides an evaluation of demographic differences in the incidence risk ratio of dengue incidence, hospitalisation and death as well as the first time-constant dengue FOI estimates, inferred by calibrating catalytic models to the age-stratified case notification data over the study period (2000–2024). This study characterises the FOI of dengue at the regional and district levels, providing critical evidence for targeting interventions and improving surveillance strategies.

**Implications of all the available evidence**

The results of this study highlight substantial regional and district-level variation in dengue transmission intensity, surveillance and reporting across Panama. These findings underscore the importance of utilising DENV transmission intensity estimates to identify high-risk areas in place of dengue incidence. Furthermore, the estimates presented in this study contribute to refine national and global dengue risk maps which in turn, can inform the design of future seroprevalence studies, resource allocation and the implementation of control interventions. Strengthening surveillance in regions with high FOI estimates and low reporting can enhance early warning systems and outbreak preparedness.

## Introduction

The increase in the incidence of dengue virus (DENV), an arbovirus transmitted by *Aedes* mosquitoes, is a major public health problem in Latin America and the Caribbean.^1^, ^2^, ^3^ In 2024, the Americas collectively reported almost 13 million cases of dengue and more than 8000 deaths, across their countries and territories.^1^ In the same year, Panama recorded the highest number of dengue cases in its history, with 32,077 case notifications, nearly double the number of cases reported in 2023 (16,577 cases).^4^ Historically, dengue has been the most prevalent vector-borne disease in Panama, with a dengue-like disease first reported in the country in 1904.^5^ The first dengue outbreaks were officially reported in 1941 and 1942.^6^ After these outbreaks, it was not until 1993 that a new dengue epidemic occurred.^7^ Of the four dengue serotypes (DENV-1, 2, 3 and 4), the most prevalent serotypes reported to be circulating in the country since 1993 are DENV-1 and DENV-2.^8^^,^^9^ Over the years, DENV-3 and DENV-4 have also been reported, in 1995 and 1998 respectively, however, they have only been detected sporadically.^8^, ^9^, ^10^ In recent years, the geographic range of arboviruses in Panama has increased significantly. This expansion is attributed to multiple factors, including the expansion of *Aedes aegypti* and *Aedes albopictus* populations, the genetic diversity of circulating DENV strains, human population growth, rapid and unplanned urbanisation, and climate change.^9^^,^^11^, ^12^, ^13^ Despite these observations, and the extensive surveillance and case recording implemented by the Ministry of Health of Panama, the epidemiology and transmission dynamics of dengue in the country are still poorly characterised.

Clinically, dengue is classified into dengue fever (DF), dengue haemorrhagic fever (DHF) and shock syndrome (DSS).^14^ Dengue infection with one serotype is believed to induce long-term immunity against that serotype, but only short-lived cross-protection against the other serotypes, meaning that individuals can potentially be infected by DENV up to four times during their lifetime.^15^^,^^16^ Epidemiological evidence indicates that secondary infections are more frequently severe, due to an immuno-pathological phenomenon called antibody dependent enhancement (ADE).^15^

Effective surveillance is essential for public health policy, to guide clinical practice and support timely disease control.^17^^,^^18^ In particular, the timeliness and accuracy of case reporting are critical for detecting and responding to outbreaks. To strengthen dengue surveillance, it is important to understand and characterise the epidemiology of dengue and to document spatial heterogeneities according to the observed data. These variations may reflect differences in healthcare access and surveillance or could be due to variations in mosquito exposure driven by heterogeneities in *A. aegypti* and *A. albopictus* abundance, which in tun can be mediated by climate, environmental and infrastructural factors, as well as demographic, and immunological factors which can all influence local transmission risk.

In epidemiology, the so-called “delay distributions” describe the probability distribution of the time between two events of clinical relevance and play a vital role in outbreak response, preparedness planning, and disease control.^18^^,^^19^ Examples of such delay distributions are the time between symptom onset and testing (“onset-to-testing”), or between symptom onset and case notification to public health agencies (“onset-to-notification”). Characterising the timeliness of surveillance in dengue endemic regions such as Panama, and in the most remote areas where connectivity and digital infrastructure are limited,^18^ is particularly important to identify differences in health care access and to inform resource prioritisation within the country.

In this work, we analysed an anonymised and unidentifiable line list dataset of laboratory confirmed DENV cases, hospitalisations and deaths from Panama spanning the last 25 years to characterise dengue epidemiology in the country. We estimated the dengue force-of-infection (FOI), which measures the annual risk of dengue infection among susceptible persons,^20^ by testing alternative hypotheses on the heterogeneity of dengue reporting across the country and assessing the evidence of age- and sex-specific trends in case reporting. The estimates generated in this study can be used to compare the risk of dengue infection across regions, characterise spatial differences in the sensitivity of disease surveillance and provide essential information to policy makers, modellers and key stakeholders in the evaluation of the potential impact of new control interventions, such as vaccination and Wolbachia.

## Methods

### Ethics statement

Ethics approval for this study has been granted by the Ethics Committee of University of Panama (Ethical Approval No. CBUP/115/2024). This study is also registered with Ministry of Health in Panama's RESEGIS (Registro y Seguimiento de Investigación para la Salud) as registration number 3740. The analysis used anonymised, routinely collected national dengue surveillance data provided by the Ministry of Health. As these data contain no personal identifiers and involve no direct contact with patients, individual informed consent was not required, in accordance with national regulations and the conditions of the issued ethical approval.

### Epidemiological and demographic data

In Panama, dengue is a notifiable disease by law, and all suspected and lab-confirmed cases must be reported to the national surveillance system. We analysed the anonymised and unidentifiable line list data of laboratory-confirmed dengue cases reported in Panama during the years 2000–2024. These data were shared by the Ministry of Health (MoH) and Instituto Gorgas de Estudios de la Salud (ICGES), which collectively manage cases surveillance in the country. Case reports are aggregated at the national level by the MoH, with laboratory confirmation supported by ICGES. Laboratory confirmation is performed using different methods, including serology (e.g., ELISA for IgM/IgG) and molecular techniques (RT-PCR), as part of the national arbovirus surveillance program. Dengue case definitions and laboratory confirmation practices in Panama changed during the study period. Until 2010, case confirmation followed the 1997 PAHO/WHO classification and relied mainly on centralised methods such as viral isolation and ELISA.^21^ After the adoption of the 2009 PAHO/WHO guidelines, case confirmation incorporated rapid tests (NS1 and IgM), and molecular techniques (RT-PCR).^22^

In Panama, the health administrative divisions include 82 districts and 16 health regions in total (Figure S1). However, although the Ministry of Health recognises the district of Arraijan as an independent health region, in the dataset used for this analysis it was consistently classified as a district within the Panama Oeste region. Therefore, throughout the study. period (2000–2024), national surveillance reports continued to aggregate dengue notifications into 15 regions.

For each reported dengue case in the study period, the dataset includes information on demographic variables (sex, age), the geographical denomination of the residence location at the administrative level 1 (health region) and 2 (district), dates of symptom onset, testing, reporting/notification, hospitalisation, and recovery.

Missing data on symptom onset were minimal; however, the completeness of the dates used to reconstruct the delay distributions varied across regions. In two indigenous regions (Guna Yala, and Ngäbe Buglé), approximately 80% of the recorded cases lacked delay information (e.g. the dates of onset, testing, notification, hospitalisation and recovery) during 2005–2024. We therefore excluded these regions from the delay distribution analysis but retained them for all other analyses where the data were complete. Given the geographically localised nature and magnitude of missingness, multiple imputation approaches were not undertaken.

The yearly incidence of dengue per 100,000 inhabitants was computed using time-varying population size estimates, stratified by age and sex for each of Panama's health region. The population estimates were obtained from the Panama MoH,^4^ which provides annual data at the district and health region levels.

The proportion of hospitalised cases was computed as the yearly number of dengue hospitalised patients divided by the yearly number of reported dengue cases. The case fatality ratio (CFR) was calculated as the number of dengue-related deaths divided by the number of reported dengue cases each year. All statistical analyses were performed using R version 4.1.3.

### Demographic differences in the incidence of dengue reporting, hospitalisation and death

We estimated demographic differences in dengue outcomes by estimating incidence risk ratios (IRR) by sex and age-group using multivariable regression. Denote $Y_{i}$ the outcome (i.e., dengue cases, hospitalisations or deaths), $N_{i}$ the population and $\Lambda_{i}$ the incidence rate of the outcome in stratum i (with reference females of age-group 0–9 years in year 2000). We assumed that the outcome was Poisson distributed with expectation $\mu_{i}$ = $N_{i}\Lambda_{i}$, i.e., that $\log\left(\mu_{i}\right)$ = $\log\left(N_{i}\right)+\log\left(\Lambda_{i}\right)\text{.}$ We estimated the incidence rate of each outcome using $\log\left(\Lambda_{i}\right)=\beta_{0}+\beta_{m}1_{m}+\sum_{k}\beta_{k}1_{k\neq 0-9}+\sum_{y}\beta_{y}1_{y\neq 2000}$ where $1_{m}$ denotes the indicator function for males, $1_{k\neq 0-9}$ the age-group indicator function for age-groups different from the reference age-group of 0–9 years, and $1_{y\neq 2000}$ is the year-specific indicator function for years >2000. The male to female IRR of each outcome was estimated using the formula shown in Equation (Eq.) (1).

Eq. 1 $$IRR_{m:f}=e^{\beta_{m}}$$

and the age-specific IRR (compared to the reference age-group of 0–9-year-olds) was estimated using the formula in Eq. (2).

Eq. 2 $$I{RR}_{k:0-9}=e^{\beta_{k}}$$

### Sensitivity analysis on the incidence of dengue reporting before and after 2010

To assess the potential effect of the different case-notification frameworks implemented since 2010 (with stricter confirmation requirements and centralised diagnostics until 2009 and decentralised rapid diagnostics introduced from 2010), we conducted a sensitivity analysis testing for demographic differences in the IRR of dengue reporting, hospitalisation and death. We fitted the same multivariable regression model described in the section “Demographic differences in the incidence of dengue reporting, hospitalisation and death” separately for the data reported in the period 2000–2009 and 2010–2024, using female, 0–9 years and respectively 2000 and 2010 as reference categories.

### Fitting probability distributions

To characterise the epidemiological delay distributions (i.e. onset-to-testing, onset-to-notification, onset-to-hospitalisation, onset-to-recovery and hospitalisation-to-recovery), we fitted five parametric probability distributions (gamma, Weibull, lognormal, logistic and Cauchy) to each delay. These statistical models were selected for their flexibility in modelling skewed frequency observations, which is typical of delay distributions.^20^

We evaluated the goodness-of-fit of each model using the Akaike Information Criterion (AIC), which is a model selection criterion that penalises the score for model complexity and where the lowest AIC value indicates the preferred model. For each delay, we fitted a statistical model based on maximum likelihood estimation (MLE) and described the summary statistics of the fitted distributions in terms of its mean, median and standard deviation using the *“fitdistrplus”* package in R.^23^

### Sensitivity analysis on the fitted probability distributions

We conducted a sensitivity analysis to evaluate whether epidemiological delay distributions varied across demographic groups and notification frameworks. First, we compared paediatric cases (≤13 years) and adult cases (≥14 years), followed by an analysis stratified by sex. Finally, to assess the potential effect of changes in dengue diagnostic and notification practices implemented since 2010, we fitted each delay separately for the pre-2010 and post-2010 periods. All analysis applied the same approach described in the section “Fitting probability distributions”.

### Mathematical models of case incidence and FOI estimates

From the line-list case-notification data, we stratified the number of dengue reported cases by region, district and year into age-groups of 9-years intervals (with $a_{j}$ and $a_{j+1}$ denoting the lower and upper bound of each age-group, for j = 1, …, 10) over a 25-year period (2000–2024).

We calibrated the catalytic model described in Imai et al.^24^ to estimate the time-constant force of infection (FOI, denoted *λ*, i.e. the per-capita per-serotype yearly risk of infection for a susceptible individual in a population). Published papers reported the circulation of mainly DENV-1 and DENV-2 in Panama, therefore we assumed that only two dengue serotypes were co-circulating in Panama and had the same probability of transmission.^9^^,^^10^

The incidence of primary infection ($I_{l,1,j}$) was modelled as shown in Eq. (3), where the integral spans all ages in the specific age group denoted by subscript j, and where $e^{-2\lambda a}$ indicates the probability of an individual of age a surviving infection to two serotypes. In the same way, the incidence of secondary infection ($I_{l,2,j})$ is given by the probabilities of having survived the infection to one serotype ($e^{-\lambda a}$), having been infected with one serotype (${1-e}^{-\lambda a}$), and the yearly risk of infection by one serotype (λ), with the 2 in front of the integral denoting the two different ways that an individual can be infected by each serotype (Eq. (4)).

Eq. 3 $$I_{l,1,j}=\int_{aj}^{aj+1}2\lambda \left(e^{-\lambda a}\right)da$$

Eq. 4 $$I_{l,2,j}=2\int_{aj}^{aj+1}\lambda \left(1-e^{-\lambda a}\right)\left(e^{-\lambda a}\right)\;da$$

The average disease incidence rate per person in age group j, denoted $D_{j}$, was computed as in Eq. (5), where $w_{j}$ represents the width of the age-group, γ is the probability that a primary infection is detected relative to a secondary infection, and *ρ* indicates the probability of a secondary infection being reported.

Eq. 5 $$D_{j}=\frac{\rho }{w_{j}}\;\left(I_{l,2,j}+\gamma I_{l,1,j}\right)$$

The number of dengue cases reported in age group *j* (C_j_) was estimated as the expected disease incidence in that age group multiplied by the population size of that age group $\left(n_{j}\right)$, as shown in Eq. (6).

Eq. 6 $$\text{Cj}=D_{j}\;n_{j}$$

We assumed that the likelihood of the total number of dengue cases follows a Poisson distribution, while the age-stratified number of cases was assumed to follow a Multinomial distribution. Inference was performed using the Hamiltonian Monte Carlo algorithm implemented in the *CmStanR* package.^25^^,^^26^

We ran 4 parallel Markov Chain Monte Carlo (MCMC) chains, each with 10,000 iterations, and 1000 warm-up steps. Convergence was evaluated visually using trace plots, and uncertainty was expressed using the 95% Credible Interval (*CrI*) of the posterior distribution of each parameter. The following prior distributions were specified for the model parameters: FOI (λ∼Normal(0,0.5)); the probability of reporting rate of secondary infection (ρ∼Normal(0.8,0.5)), the probability of reporting a primary infection relative to a secondary infection (γ∼Normal(0.8,0.5)).

The model was implemented at both the health region and district level, estimating health region-specific and district-specific FOIs, and national or health region-specific parameters for the γ and ρ.

### Sensitivity analysis: regional and age-dependencies in dengue reporting

In the baseline model we assumed homogeneous dengue surveillance and reporting across the country by fitting the model defined in Eqs. Eq. 3, Eq. 4, Eq. 5, Eq. 6 at the regional level, estimating a unique reporting rate (ρ) across all regions of Panama (**Model A**). In **Model B** we assessed potential spatial heterogeneities in reporting across the country by testing the extent to which the data support region-specific reporting rates ρ by allowing this parameter to vary by region.

In a further sensitivity analysis, we explored age-dependent differences in reporting, and extended Models A and B by introducing a scaling factor (*k ∼* Normal (0, 1)), to adjust the reporting rate ρ of the 0–9 years age-group (**Models A1** and **B1**) or the 10–19 years age-group (**Models A2** and **B2**).

Model performance and selection (between models A1, A2, B1 and B2) was evaluated using the Widely Applicable Information Criteria (WAIC).^27^^,^^28^ We then used the best performing model at the health region-level to generate district-level FOI estimates.

### Proportion seropositive at 9 years of age

The expected seroprevalence at age 9 years, denoted $S_{9}$, was computed using the estimated per-serotype FOI λ as shown in Eq. (7).

Eq. 7 $$S_{9}={1-e}^{-2\lambda \times 9\;}$$

### Sensitivity of dengue surveillance

To assess the effectiveness of dengue surveillance systems across different regions, we estimated the sensitivity of surveillance (*SS*_*l*_) for each location as the ratio of the total number of reported dengue cases divided by the estimated number of primary and secondary infections across all age groups over the study period, as shown in Eq. (8).

Eq. 8 $$SS_{l}=\frac{\sum_{j}C_{l,j}\;}{\sum_{j}\;\left(I_{l,1,j}+I_{l,2,j}\right)\times N_{l,j}\;}\times 100$$

In this formulation, the terms $I_{l,1,j}$ and $I_{l,2,j}$ represent the estimated incidence of primary and secondary infections, respectively, for age group j in location l. The variable $N_{l,j}$ denotes the population size for age group j and location l, while $C_{l,j\;}$ indicates the number of observed dengue cases in age group j and location l.

### Sensitivity analysis on the reporting rate before and after 2010

To evaluate the potential effect of the 2010 changes in dengue case definitions, diagnostic workflows, and reporting procedures on the FOI estimates, we conducted a sensitivity analysis in which the reporting rate was estimated separately for the periods 2000–2009 and 2010–2024. We fitted the catalytic model described Eqs. Eq. 3, Eq. 4, Eq. 5, Eq. 6) independently to both time periods, using the structure described for Model B2.

### Role of funding source

The funders had no role in the study design, data collection, data analysis, data interpretation, or writing of the manuscript.

## Results

### Epidemiological and demographic data

**Fig. 1:**
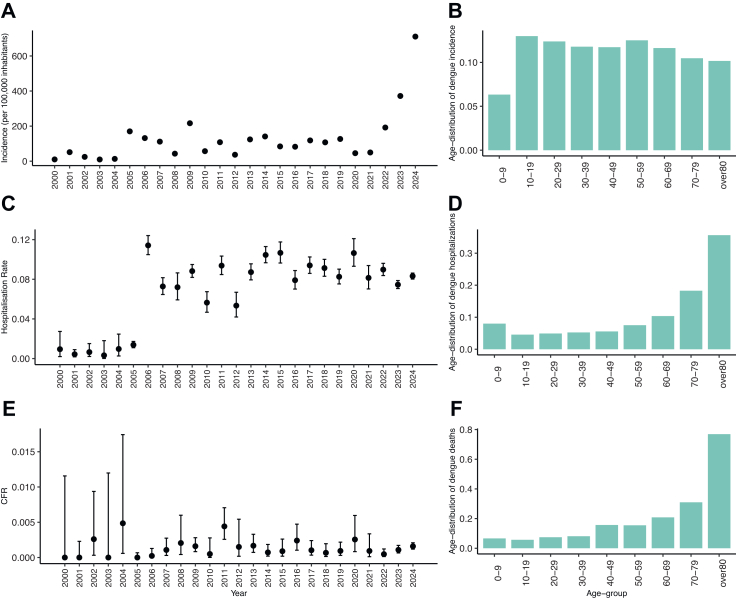
Epidemiology of dengue in Panama. (A) Annual dengue incidence (per 100,000 inhabitants) and (B) age-distribution of notified dengue cases reported over the entire study period (2000–2024). (C) Annual mean and 95% Confidence Intervals (CI), hospitalisation rate (computed as the yearly number of hospitalised patients divided by the yearly number of dengue reported cases) and (D) age-distribution of the number of hospitalised cases over the study period (2000–2024). (E) Mean and 95% CI, Case Fatality Ratio (CFR, computed as the yearly number of dengue-attributable deaths divided by the yearly number of dengue cases reported) and (F) age-distribution of the reported dengue deaths over the study period (2000–2024).

Dengue incidence was 710 per 100,000 inhabitants in 2024, which represents an increase of 91% compared to 2023 (372 per 100,000 inhabitants), and marking the highest dengue incidence reported in Panama since 2000 (Fig. 1A). Hospitalisation rates varied across the study period (2000–2024), peaking in 2006 at 11.4%, with the lowest rate observed in 2003 (0.30%) (Fig. 1C). The CFR remained low overall, with the highest recorded in 2004 (0.49%) and no dengue-attributable deaths reported in 2000, 2001, 2003 and 2005 (Fig. 1E). Whilst we do not observe marked changes in dengue incidence and in hospitalisation rates and CFRs before and after 2010, we cannot exclude that changes in case diagnosis and confirmation influenced the observed temporal trends. When comparing the age-distribution of the notified dengue cases before and after 2010, we observe that the introduction of rapid diagnostic tests and decentralised testing increased the incidence of reported dengue in the younger age-groups in 2010–2024 (Figure S2).

**Fig. 2:**
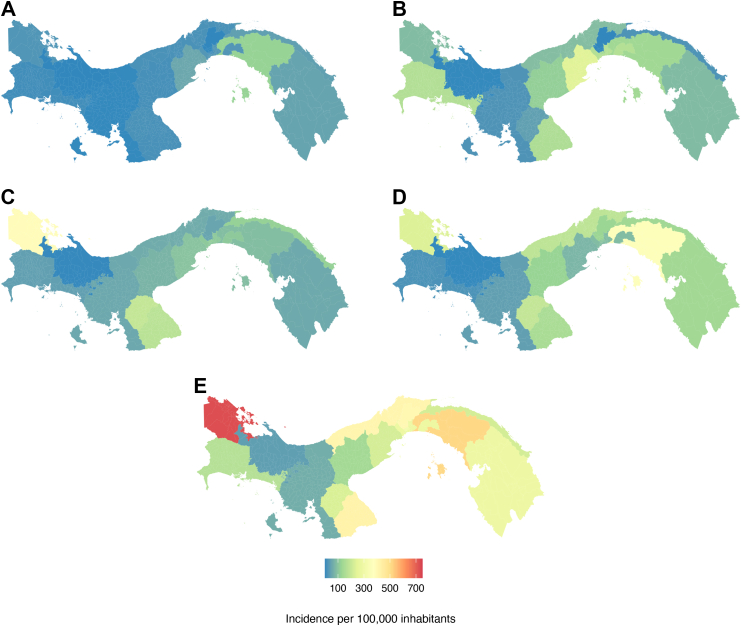
Average incidence of dengue by region over time. The maps show five-year intervals: (A) 2000–2004, (B) 2005–2009, (C) 2010–2014, (D) 2015–2019, (E) 2020–2024. The colour scale indicates the average yearly incidence per 100,000 inhabitants.

The incidence of dengue across the different regions shows an overall increase in recent years (Fig. 1). The regions with the highest incidence per 100,000 inhabitants between 2000 and 2024 were Bocas del Toro (1937 per 100,000 inhabitants) in 2023 followed by Los Santos (1400 per 100,000 inhabitants) in 2024. Regions such as Veraguas and Chiriquí experienced relatively stable incidence rates over the study period, while others, such as Panama Este, and Colón, experienced a marked increase in the incidence since 2015 and 2020 (Fig. 2), several years after the changes in dengue surveillance implemented since 2010, which may suggest actual increases in dengue transmission intensity. The extent to which the observed increases in dengue incidence can be attributed to changes in surveillance versus transmission will need to be investigated in future studies.

### Demographic differences in dengue reporting

**Fig. 3:**
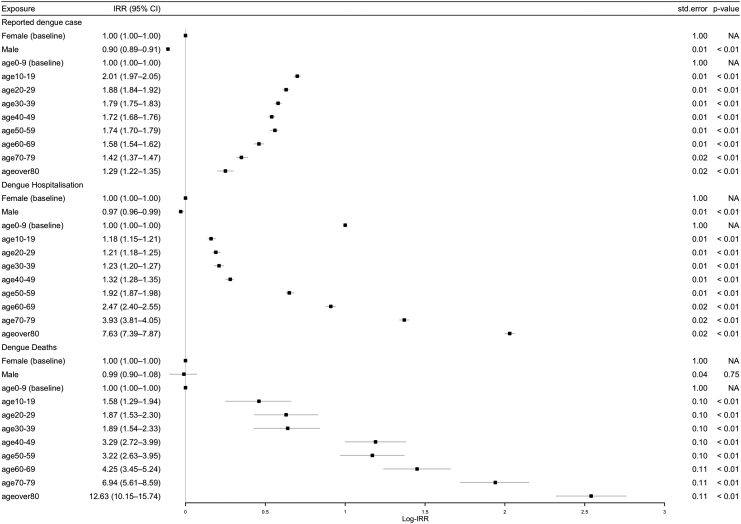
Incidence risk ratio (IRR) for dengue cases, hospitalisations and deaths, estimated using multivariable regression, by age-group and sex for whole study period (2000–2024). For each exposure group, the figure shows the mean of the Log-IRR and its 95% Confidence Interval (CI), the standard error of the IRR (std.error) and the p-value (p value). The reference category was female, 0–9 age group in 2000. The year-specific IRRs are not shown.

The sex-specific dengue incidence estimates suggest that, at the national level, males are less likely to report a dengue infection than females (IRR = 0.90, 95% CI [0.89–0.91], p < 0.0001) (Fig. 3). We also found that the propensity of reporting a dengue infection in individuals aged 10 years and above was higher than the 0–9 years reference group (p < 0.0001) (Fig. 3).

The dengue cases reported across all age groups tended to have a significantly higher risk of hospitalisation as compared to the reference age group of 0–9 year olds, with the highest risk observed for the age groups 60–69 years, 70–79 years and 80 years and above (respectively, IRR = 2.47, 95% CI [2.40–2.55], IRR = 3.93, 95% CI [3.81–4.05] and IRR = 7.63, 95% CI [7.39–7.87], p < 0.0001) (Fig. 3).

The highest proportion of dengue attributable deaths reported in Panama was 0.49% in 2004 (5 per 1000 cases) (Fig. 1). Over the study period, the incidence of death indicates no significant difference between males and females (IRR = 0.99, 95% CI [0.90–1.08], p = 0.75). However, we found that the incidence of death was significantly higher in all age-groups compared to the reference age-group of 0–9 year olds, with the highest incidence risk ratio estimated in the age group over 80 years as compared to the reference age-group of 0–9 year olds (IRR = 12.63, 95% CI [10.15–15.74], p < 0.0001) (Fig. 3). The results of the sensitivity analysis on the IRR of dengue reporting hospitalisation and death for the time periods 2000–2009 and 2010–2024 are presented in Table S1.

### Fitted probability distributions

**Fig. 4:**
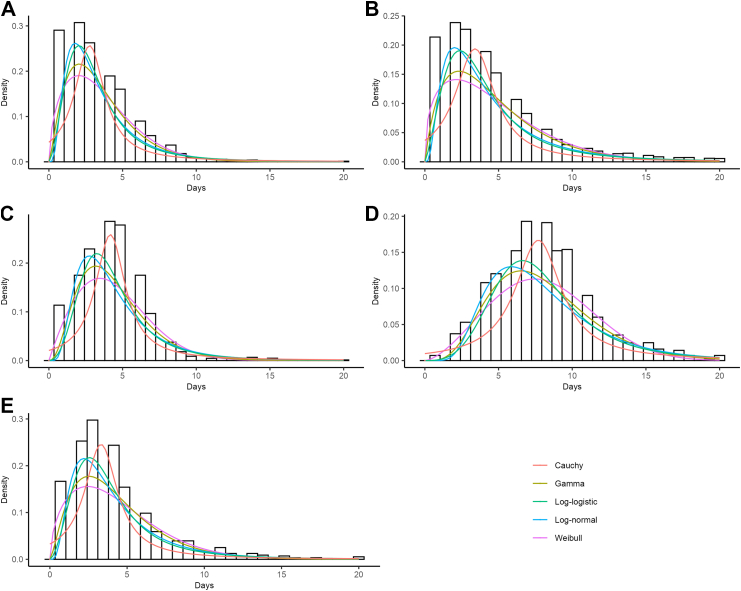
Distribution of the observed national delay data reported over the time period 2000–2024. Distribution of (A) symptoms onset-to-testing; (B) symptoms onset-to-reporting; (C) symptoms onset-to-hospitalisation; (D) symptoms onset-to-recovery; and (E) hospitalisation-to-recovery. The bars represent the observed data. The lines indicate the fitted probability density functions, including the Cauchy distribution (red), Gamma distribution (yellow), Loglogistic distribution (green), Lognormal distribution (blue), and Weibull distribution (purple).

We found that the observed onset-to-testing, onset-to-reporting, and hospitalisation-to-recovery delay distributions were best reconstructed through a lognormal distribution. The gamma distribution fitted more closely the observed onset-to-hospitalisation delay, while the Weibull distribution was selected for the onset-to-recovery delay (Fig. 4). The AIC of each delay fitted at the national and regional level are shown in Table S2.

From the fitted distribution, we estimate that, at the national level, the time between symptoms onset-to-testing was on average 3.48 days (95% CI: 3.39–3.57), the time between symptoms onset-to-reporting was on average 4.78 days (95% CI: 4.72–4.84), that hospitalised cases were admitted on average 4.49 days (95% CI: 4.22–4.76) from symptoms onset, that hospital discharge occured on average 7.82 days (95% CI: 6.47–8.85) from symptoms onset and that the time from hospitalisation to recovery was on average 4.16 days (95% CI: 3.26–4.52) (Table S3).

**Fig. 5:**
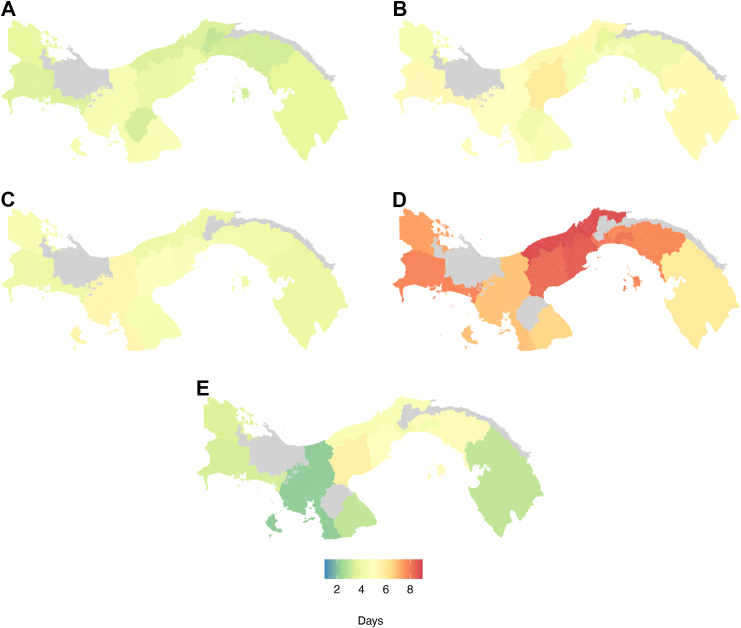
Median of the fitted delay distribution by region for the time from: (A) symptoms onset-to-testing; (B) symptoms onset-to-reporting; (C) symptoms onset-to-hospitalisation; (D) symptoms onset-to-recovery; and (E) hospitalisation-to-recovery. The regions are shown in a colours scale to reflect the median delay in days, with a colour scale ranging from blue, indicating low delay to red, indicating high delay. Regions (Guna Yala and Ngäbe Buglé and Herrera) in grey indicate insufficient data to estimate the delay distribution.

We observed evidence of spatial differences in the average delay distributions by health regions, reflecting differences in the dispersion and central tendency of the delays. In Panama Norte, for example, dengue testing occurred on average 3.04 days (95% Crl: 2.95–3.14) after symptoms onset, whereas in Los Santos testing tended to occur 4.63 days (95% Crl: 4.44–4.82) after symptoms onset (Table S3, Fig. 5).

We also found heterogeneity in the timeliness of dengue reporting across Panama. Cocle showed the longest median onset-to-reporting delay, with a median of 5.96 days (95% Crl: 5.79–6.12) (Table S3, Fig. 5). On the other hand, in the Panama Norte region, we observed the shortest median onset-to-reporting delay of 2.80 days (95% Crl: 2.58–3.02) (Table S3, Fig. 5).

Our result show that also the delay between symptom onset and hospitalisation varied by regions, with Veraguas exhibiting the longest median delay of 5.44 days (95% Crl: 4.71–6.18). In contrast, Darien had the shortest median time from symptoms onset-to-hospitalisation of 3.51 days (95% Crl: 3.47–3.56).

Colon region tended to have the longest median onset-to-recovery delay, with an average of 8.75 days (95% Crl: 7.92–9.58) (Table S3). The time from hospitalisation-to-recovery also varied substantially across the country, with Cocle showing the longest time with a median of 5.86 days (95% Crl: 3.70–8.02). The shortest duration of hospitalisation was observed in Veraguas with a median of 2.17 days (95% Crl: 0.75–3.58).

We also assessed the evidence around potential differences in the observed delays between the paediatric age groups (≤13 years) and adults (≥14 years). The median delay times are generally shorter in the paediatric group compared to older individuals (Table S3). For example, the onset-to-recovery time in the paediatric population was on average 3.56 days (95% Crl: 2.96–4.16), compared to 8.14 days (95% Crl: 7.90–8.38) in adults. These differences are further illustrated in Figure S3.

Additionally, we found no significant differences in the time between symptoms onset and reporting between females and males (median onset-to-reporting delay for males was of 4.39 days (95% Crl: 4.31–4.47) compared to 4.45 days (95% Crl: 4.37–4.54 for females). The fitted distributions by region are summarised in Table S3.

The statistics of the delay distributions obtained in the sensitivity analysis for the time periods pre-2010 and post-2010 are presented in Table S4, which show a significant reduction in the onset-to-reporting delay since the implementation of the case confirmation protocol from 2010 onwards.

### Dengue transmission intensity in Panama

**Fig. 6:**
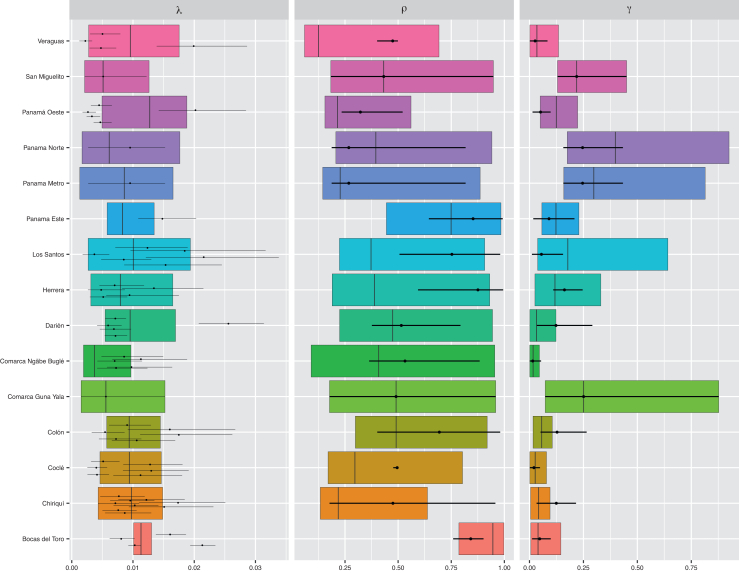
Constant-in-time per-serotype FOI (λ), secondary infections (ρ), and relative reporting rate of primary versus secondary infections (γ) estimated by region (boxplot). For each region, the boxplots show the median estimates (bar), and the box represents the 95% credible intervals (Crl) of the estimates obtained from the fit of the selected model (model B with scaling factor for the 10–19 age-group) to the regional case data. Estimates at the district-level are also reported for λ, ρ and γ as points (media) and error bars representing their 95% CrI (having assumed that all districts within the same region share the same reporting rates).

Model version B2, with a scaling factor modifying the reporting rates for the age group 10–19 years, was selected as the best model according to the WAIC (Table S5). Therefore, below we present the estimates obtained with this model version. At the regional-level, the estimated per-serotype FOI (λ) ranged from 0.004 (95% CrI: 0.002–0.010) in Ngäbe Buglé to 0.013 (95% CrI: 0.005–0.019) in Panama Oeste. Most regions had a median per-serotype FOI (λ) between 0.006 and 0.011 (Fig. 6, Table S6).

**Fig. 7:**
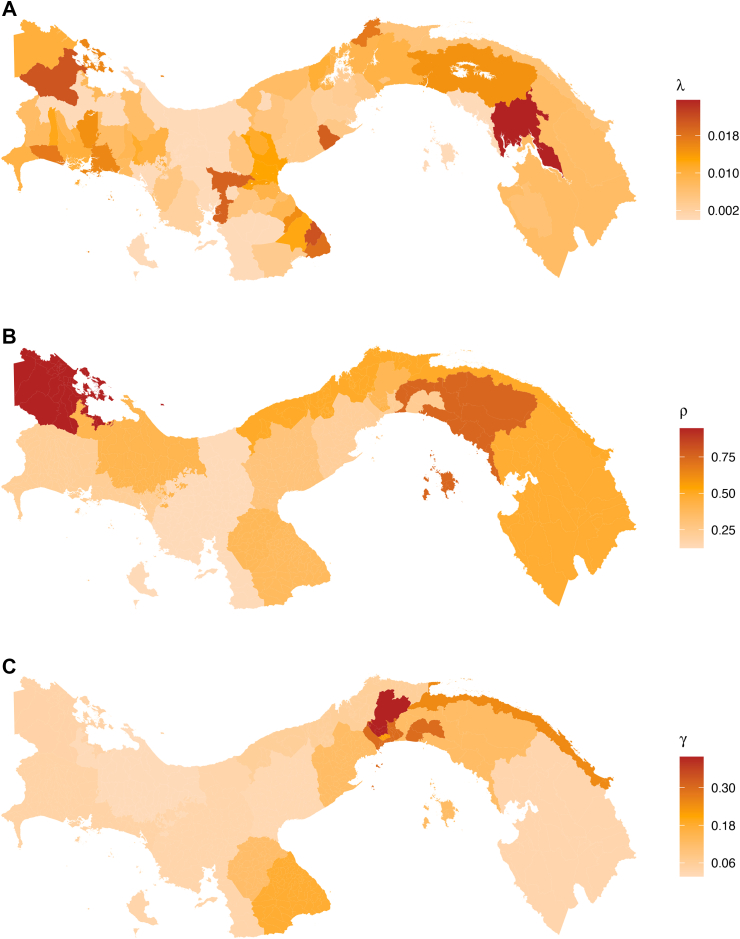
Average model parameter estimates across the Panamanian districts obtained from the fit of the best model (Model B with scaling factor for the reporting rate of 10–19-year-olds) to the dengue cases notified during the study period (2000–2024). A. district-level constant-in-time per-serotype λ, B. the region-level probability of a secondary infection being reported (ρ), and C. region-level probability that a primary infection is detected relative to a secondary infection (γ).

At the district level, we estimated a dengue FOI ranging from an average of 0.002–0.026. The highest district-level λ was estimated for Santa Fé (Darien region) and Pocri (Los Santos region) with a median of 0.026 (95% CrI: 0.021–0.031) and 0.022 (95% CrI: 0.012–0.034), respectively. A similar estimate was obtained also in the district of Almirante (0.021, 95% CrI: 0.019–0.023) (Bocas del Toro region). The districts presenting the lowest FOI were Capira (Panamá Oeste Region) [0.003 (95% CrI: 0.002–0.004)] and Soná (Veraguas Region) [0.002 (95% CrI: 0.001–0.003)] (Fig. 7A, Table S7).

We assessed spatial autocorrelation in the FOI estimates using Moran's l with contiguity-based spatial weights matrix. As shown in Table S10, the Moran's I diagnostics indicated no significant global spatial autocorrelation at either the regional or district level (Region: I = −0.05, p = 0.46, z = 0.11; District: I = 0.09, p = 0.14, z = 1.07). These results suggest that the observed spatial heterogeneities in FOI reflect actual spatial differences rather than spatial clustering (Table S8).

The reporting probability of a secondary infections (*ρ*) showed large regional variation (Fig. 7). Bocas del Toro had the highest *ρ* estimate, with a median of 0.946 (95% CrI: 0.786–0.998). Conversely, Veraguas had the lowest *ρ* estimates, with median of 0.125 (95% CrI: 0.059–0.692), indicating large under-reporting of secondary infections. In urbans areas such as Colon and Panama Este we estimate moderate to high reporting of secondary infections (respectively, 0.491, 95% CrI: 0.299–0.919 and 0.794, 95% CrI: 0.445–0.984) (Fig. 6, Table S6).

The probability of detecting primary infections relatively to secondary infections (ρ) also largely varied across regions (Fig. 7). Panama Norte exhibited the highest γ estimate, with a median of 0.398 (95% CrI: 0.175–0.925) indicating that the detection of primary infections is about half that of secondary infections. Ngäbe Buglé and Cocle presented the lowest γ estimates, with a median of 0.017 (95% CrI: 0.001–0.461) and 0.026 (95% CrI: 0.002–0.079) respectively (Fig. 6, Table S6), indicating lower probabilities of detecting primary infections relatively to secondary infections.

All details on the model fit and parameter estimates at regional- and district-level are presented in Figures S4 and S7 and Tables S6 and S7 of the Supplementary Information.

**Fig. 8:**
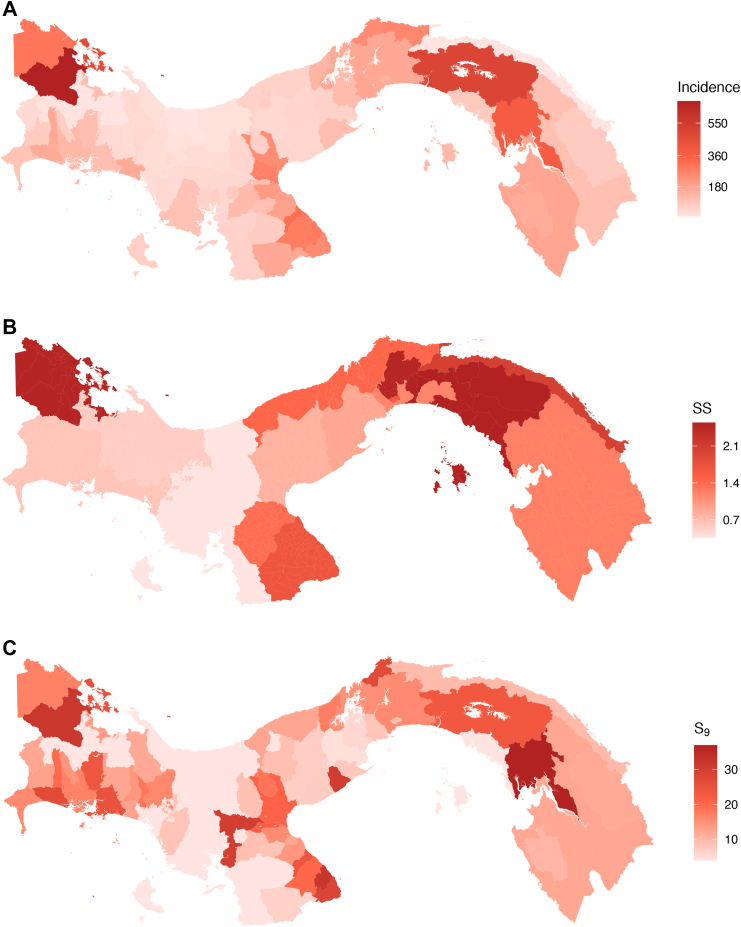
Dengue-related indicators across Panama based on reported cases during the study period (2000–2024). A. Average annual incidence of dengue disease by districts (incidence per 100,000 inhabitants); B. Sensitivity of dengue surveillance (SS) by region; C. Seroprevalence estimates (expressed as %) at 9 years of age $\left(S_{9}\right)$ by districts.

The expected seroprevalence at 9 years of age (Fig. 8C), based on WHO recommendations for assessing dengue endemicity,^29^ and reconstructed from the FOI estimates, was below 40% across the whole country. The highest estimate was observed in Santa Fé district, located in the Darien region [median 36.9% (95% CrI: 31.1%–43.1%)].

The estimated sensitivity of dengue surveillance varied considerably across regions in Panama (Fig. 8B and Table S10). The highest sensitivity was observed in Panama Este (SS = 2.54%, 95% CI: 1.79%–3.39%), Bocas del Toro (SS = 2.50%, 95% CI: 2.25%–2.72%), and Panama Norte (SS = 2.48%, 95% CI: 1.18%–7.86%). In contrast, regions such as Veraguas (SS = 0.35%, 95% CI: 0.24%–0.99%), Ngäbe Buglé (SS = 0.56%, 95% CI: 0.24%–1.04%), and Chiriqui (SS = 0.64%, 95% CI: 0.48%–1.23%), showed notably lower proportions of notified cases among the estimated infections. The results of the sensitivity analysis conducted to assess the effect of the 2010 changes in dengue case definitions, diagnostic workflows, and reporting procedures. The results, including posterior predictive check and full model performance statistics, are provide in Table S10.

### Data and code availability statement

Data and code for reproducing the results of this study can be accessed at https://github.com/mrc-ide/Dengue-Epidemiology-and-Transmission-Intensity.git.

## Discussion

This study characterises the epidemiology of dengue and the intensity of dengue transmission across Panama over the period 2000–2024 using a national dataset of laboratory confirmed dengue cases. We focused on laboratory-confirmed cases to ensure diagnostic accuracy and reduce misclassification, as recommend by PAHO/WHO guidelines.^30^ In Panama, the national incidence of reported dengue cases has been steadily rising throughout the years, peaking at 720 per 100,000 inhabitants in 2024. The geographical distribution of the reported dengue cases is highly variable across the country, with regions such as Bocas del Toro and Los Santos reporting an incidence of 1935 and 1400 cases per 100,000 inhabitants respectively in 2023 and 2024, both representing the highest incidences observed in the past 25 years. In contrast, the lowest dengue incidences were reported in regions such as Chiriqui and Veraguas in 2000, with 0.2 and 0.4 cases per 100,000 inhabitants, respectively.

Our estimates show longer onset-to-reporting delays in rural regions such as Cocle, compared to those observed in typical urban settings such as Panama Norte, where access to health care tends to be easier and where shorter delay in case reporting are also observed. Similarly, the analysis of the onset-to-testing delay reveals substantial regional variations. The most prolonged delays were found in Los Santos, suggesting potential challenges in timely diagnostic access in this health region. In contrast, shorter delays were recorded in Panama Norte, indicating more efficient health services. These heterogeneities suggest differences in healthcare infrastructure across the country, which likely lead to disparities in disease detection and case reporting.^8^

We observed higher hospitalisation rates in the age groups over 50 years, which suggests the higher vulnerability of older adults to severe dengue symptoms and may reflect antibody decay, decreased immunity, the presence of comorbidities or age-dependencies in hospitalisation practices as suggested in other studies.^31^, ^32^, ^33^

Using catalytic models to characterise dengue transmission intensity, we found high heterogeneity in the per-serotype FOI and reporting rates across regions and districts. Whilst most regions had an average per-serotype FOI between 0.004 and 0.015, this analysis identified hotspots of transmission in Panama Oeste, a region not previously identified as a high-risk area, where we estimated the highest FOI in the whole country (average per-serotype FOI of 0.009). At the district level, Santa Fe (Darien region) and Pocri (Los Santos region) had the highest per-serotype FOI (average of 0.024), followed by Almirante (Bocas del Toro region), and San Carlos (Panama Oeste region) with an average per-serotype FOI of 0.021. Conversely, we estimated that the population of Soná (Veraguas Region) and Capira in Panama Oeste region had a lower per-serotype FOI with an average of 0.005. Importantly, several districts exhibited substantially higher and lower FOIs than their regional averages, highlighting how hotspots of transmission can be focal and that analyses conducted at low spatial resolution can smooth heterogeneities in risk (Fig. 6). These results underscore the importance of investigating dengue transmission at the finest spatial resolution possible to capture such heterogeneity, which may be masked at broader administration levels. In future work, it will be important to investigate the climate, environmental, demographic, social and economic drivers behind these spatial differences in infection risk, e.g. linked to agricultural and livestock farming activities, which can potentially create favourable conditions for mosquito breeding.^34^^,^^35^

These estimates of dengue FOI offer practical guidance for public health planning. For example, areas with high FOI indicate where strengthening of surveillance or intensified vector control may be prioritised. As FOI estimates can be translated into seroprevalence estimates, these help to identify communities with sustained high exposure or, on the other hand, greater risk of future outbreaks. As FOI estimates can be directly compared between locations (differently from incidence estimates), the estimates generated in this study can be translated directly into operational decisions on where, when, and how to allocate public health resources. In our analysis we found that the estimated seroprevalence at 9 years in Panama is below 40% across all regions, indicating that the implementation of a dengue vaccination programme using the TAK-003 vaccine is not recommended according to the current WHO vaccine guidelines.^29^

Comparing our estimates with global trends, Panama's per-serotype FOI estimates (approximately 0.004–0.015 at the region-level; with local hotspots reaching up to 0.024 at the district-level) fall within the lower to moderate range of the FOIs observed worldwide.^36^^,^^37^ In the Americas, FOI estimates in hyperendemic regions such as Brazil and Colombia are substantially higher that those observed in Panama.^36^^,^^37^ Thus, while Panama exhibits marked spatial heterogeneity and localised hotspot, its overall transmission intensity is currently lower than that of hyperendemic regions in parts of Latin America and Southeast Asia. Going forward, it will be useful to monitor the extent to which this trend may persist or change due to climate change.

Placing Panama's FOI within this global and regional context underscores the importance of evaluating local surveillance performance, as differences in reporting sensitivity can mask or amplify apparent transmission patterns. Our estimates suggest substantial spatial heterogeneity in the capacity of the local surveillance system to detect and report infections across the country. Regions such as Panama Este and Bocas del Toro exhibited higher sensitivities (approximately 2.5% on average) and higher incidence (Fig. 8A) as compared to regions such as Veraguas and Ngäbe Buglé where the sensitivity of surveillance was low (on average < 0.5%) despite the reporting of moderate incidence rates, which indicates gaps in case detection and reporting capacity. The comparison between case incidence, seroprevalence and the sensitivity of surveillance (Fig. 8) highlights the value of using indicators such as the FOI, which provide comparable measures of transmission intensity and infection risk having accounted for differences in reporting.

This study has limitations. First, the individual-level data on the dates of onset, testing, notification, hospitalisation and recovery were largely incomplete in the indigenous health regions of Guna Yala and Ngäbe Buglé, which indicates that reporting should be strengthened and that national-level estimates should be interpreted with caution in these local contexts. Second, the epidemiological delay distributions were modelled at the population level assuming independence, whilst at the individual-level epidemiological delays are not necessarily independent; the extent to which this assumption may underestimate the uncertainty remains to be explored in future work. Third, this analysis assumes the circulation and equal transmissibility of two serotypes, given the evidence of DENV-1 and DENV-2 circulation across the country. This was done because across the entire period analysed, most of the dengue cases with serotype information determined by PCR were identified as DENV-1 or DENV-2, with no evidence of sustained transmission of the other serotypes. Based on these observations, we assumed endemic circulation of only DENV-1 and DENV-2. We acknowledge that the two serotypes may not circulate with equal intensity each year, but this assumption was consistent with previous studies.^9^^,^^10^ Having robust data on circulating serotypes is key to estimate the immunity profile of the population against the different serotypes, and in future analyses it will be interesting to relax the assumption of equal transmissibility of the dengue serotypes. Fourth, a limitation of this study lies in the available surveillance data, as routinely collected dengue case data do not distinguish primary and secondary infections. Strengthening the national dengue surveillance system to systematically capture serological evidence of primary or post-primary infection would enable improved case classification and enhance the value of future epidemiological and modelling studies of dengue transmission.

In addition, interpretation of dengue surveillance data is constrained by significant change in case definitions, diagnostic practices, and reporting over study period, specially around the 2010 national surveillance system update. From 2000 to 2009, Panama applied the 1997 PAHO/WHO classification,^21^ where suspected cases were defined by fever plus two or more symptoms and epidemiological link, and severity was categorised as dengue fever (DF), dengue haemorrhagic fever (DHF), and dengue shock syndrome (DSS). After the 2009 PAHO/WHO revision, Panama adopted the update classification into dengue without warning signs, dengue with warming signs, and severe dengue. Diagnostic confirmation also evolved: prior to 2010, confirmation relied on centralised, hight-specified methods such as viral isolation and ELISA, whereas after the adoption of PAHO/WHO 2009 guidelines,^22^ and the introduction of rapid diagnostic tests (NS1 and IgM), confirmation became decentralised. Molecular tools such as multiplex RT-PCR were progressively implemented but remained limited compared to rapid test. Whilst these changes may have biased the proportion of infections reported in the pre- and post-2010 period due to stricter confirmation criteria, in our sensitivity analysis assessing the impact of time-varying reporting rates (before and after 2010), we did not identify large changes in FOI (Table S10). Future analyses of dengue time series, disease dynamics, and time-varying FOI estimates should also account for the changes in dengue surveillance occurred since 2010, and for the co-circulation of other arboviruses (e.g., Zika, Chikungunya, Punta Toro, Oropuche) with overlapping clinical presentation, which may have increased the risk of misclassification during the periods of co-circulation. Notably, the extent to which the increasing trends in dengue incidence can be explained by changes in the surveillance system (e.g., changes in the case definition and laboratory confirmation methods introduced since 2010) versus actual changes in the intensity of dengue transmission, potentially driven by the changing climate, is subject of future investigations.

In conclusion, our study provides evidence of regional and district-level variability in dengue reporting, surveillance and transmission intensity across Panama. These findings have practical implications for dengue surveillance, prevention and control, as regional- and district-level differences in the distribution of epidemiological delays shed light on the past and recent responsiveness of dengue surveillance, and the FOI and seroprevalence estimates uncover the hidden burden of dengue infection in Panama. The findings presented in this study show that the incidence of reported cases is affected by differences in healthcare provision and access across the country. By identifying areas with sustained dengue transmission despite low reported incidence, our results support a shift from reliance on reported cases alone toward risk-informed surveillance strategies and help identify priority settings in which to target new interventions aimed at reducing dengue transmission and hospitalisations. As such, the results presented in this paper can be used by the national and local governments to prioritise local investments in dengue diagnostics, surveillance and control to ultimately reduce the health and economic burden of dengue in the country.

## Contributors

Data acquisition: DL, RR, FB, LC, LM, YD; Data curation: MQ; Formal analysis: MQ, AV, ID; Methodology: MQ, AV, ID; Software: MQ, AV, ID; Validation: MQ, AV, ID; Visualisation: MQ, AV, ID; Writing- Original Draft; Preparation: MQ, AV, ID; Writing. review & editing: MQ, AV, ID, DL, RR, FB, LC, LM, YD; Funding acquisition: MQ, ID; Supervision: AV, ID; Conceptualisation: MQ, AV, ID, DL.

## Data sharing statement

The anonymised dengue surveillance data used in this study were provided by the Ministry of Health of Panama and Instituto Conmemorativo Gorgas estudios de la Salud. Access to these data required approval from Ethics Committee of University of Panama (Ethical Approval No. CBUP/115/2024) and registration with Ministry of Health in Panama's RESEGIS (3740). The code and data used in this study are available at https://github.com/mrc-ide/Dengue-Epidemiology-and-Transmission-Intensity.git.

## Editor note

The Lancet Group takes a neutral position with respect to territorial claims in published maps and institutional affiliations.

## Declaration of interests

All the authors declared no competing interests.
