# Supplementary material for: Dengue epidemiology and transmission intensity across Panama during 2000–2024: a modelling study
Source: Lancet Reg Health Am. 2026 Apr 22;58:101471. doi: 10.1016/j.lana.2026.101471 (PMC13123576; doi:10.1016/j.lana.2026.101471)
Supplement: Supplementary [file mmc1.docx]

Supplementary Information

**Dengue epidemiology and transmission intensity across Panama during 2000-2024. A modelling study.**

Mario Quijada^1,2,^^, Anna Vicco^1^, Fulvia Bajura^3^, Lourdes Moreno^3^, Yamilka Diaz^4^, Daniel Laydon^1^, Lizbeth Cerezo^3^, Reina Roa^3^*, Ilaria Dorigatti^1^*^

Department of Infectious Disease Epidemiology, School of Public Health, Faculty of Medicine, Imperial College London, London, United Kingdom; 2. Estación Biomédica Experimental, Instituto Conmemorativo Gorgas de Estudios de La Salud, Panamá; 3. Ministerio de Salud de Panamá, Panamá; 4. Departamento de Investigación en Virología y Biotecnología, Instituto Conmemorativo Gorgas de Estudios de La Salud, Panamá. * Shared senior authorship.

^Corresponding author: [m.quijada22@imperial.ac.uk](mailto:m.quijada22@imperial.ac.uk), [i.dorigatti@imperial.ac.uk](mailto:i.dorigatti@imperial.ac.uk)

Table of Contents

[1. Figures 2](#_Toc219715612)

[2. Tables 7](#_Toc219715618)

### Figures


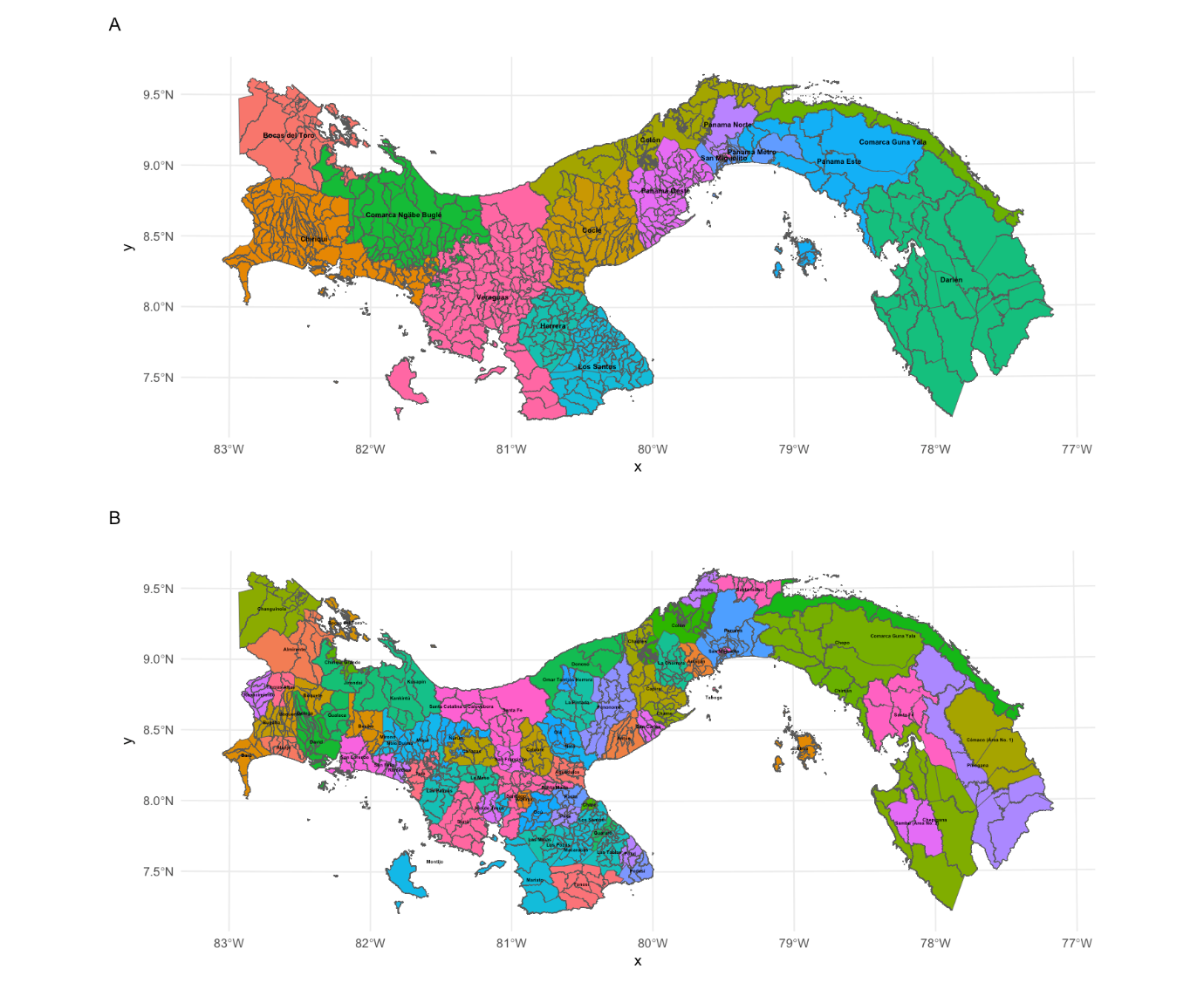


**Figure S1**. Administrative divisions of the Republic of Panama at two levels. A. Health Region-level map: the administrative division at the health region level, each region is represented by a distinct colour; B. District-level map: each district is shown in a different colour.


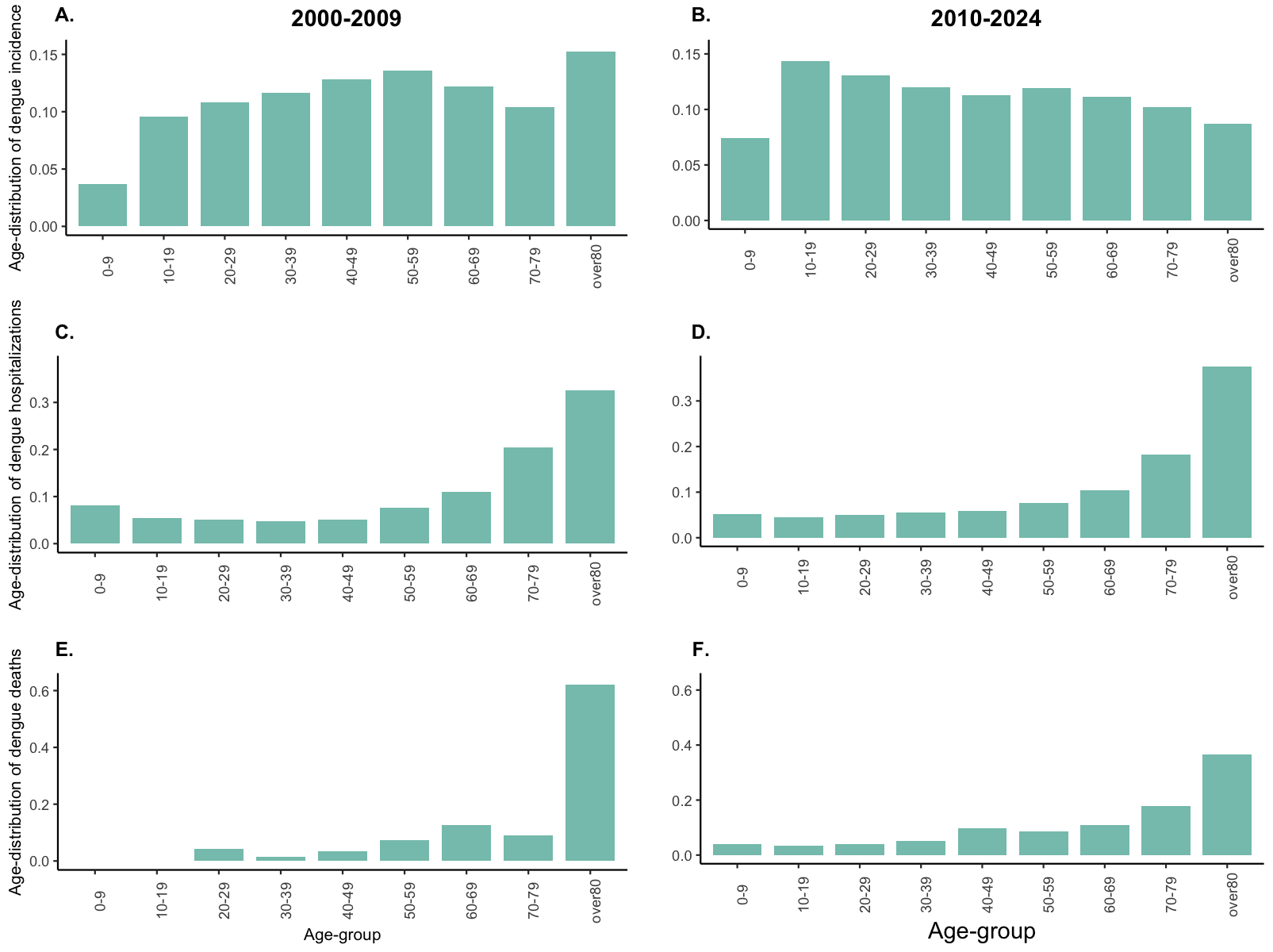


**Figure S2**. Epidemiology of dengue in Panama by age-group for two periods: before 2010 and from 2010 onward. Age-distribution of the dengue incidence reported over the time period (A) 2000-2009 and (B) 2010-2024. Age-distribution of the number of hospitalised cases reported over time period (C) 2000-2009 and (D) 2010-2024. Age-distribution of the reported number of dengue deaths in the time period (E) 2000-2009 and (F) 2010-2024.

*
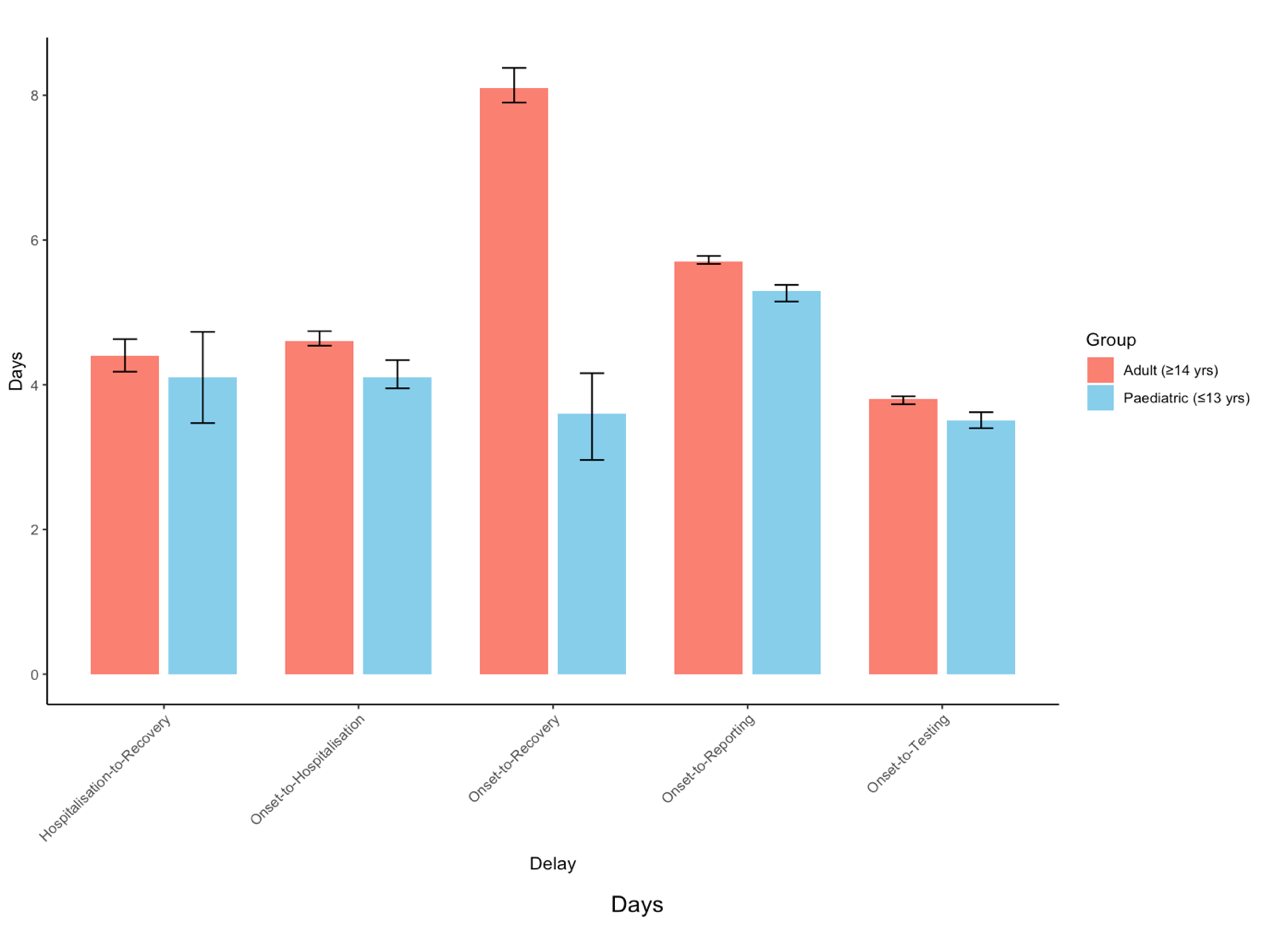
*

**Figure S3**. Median delay times and 95% CI (error bar) of the paediatric (blue) and adult (red) cohorts (group).


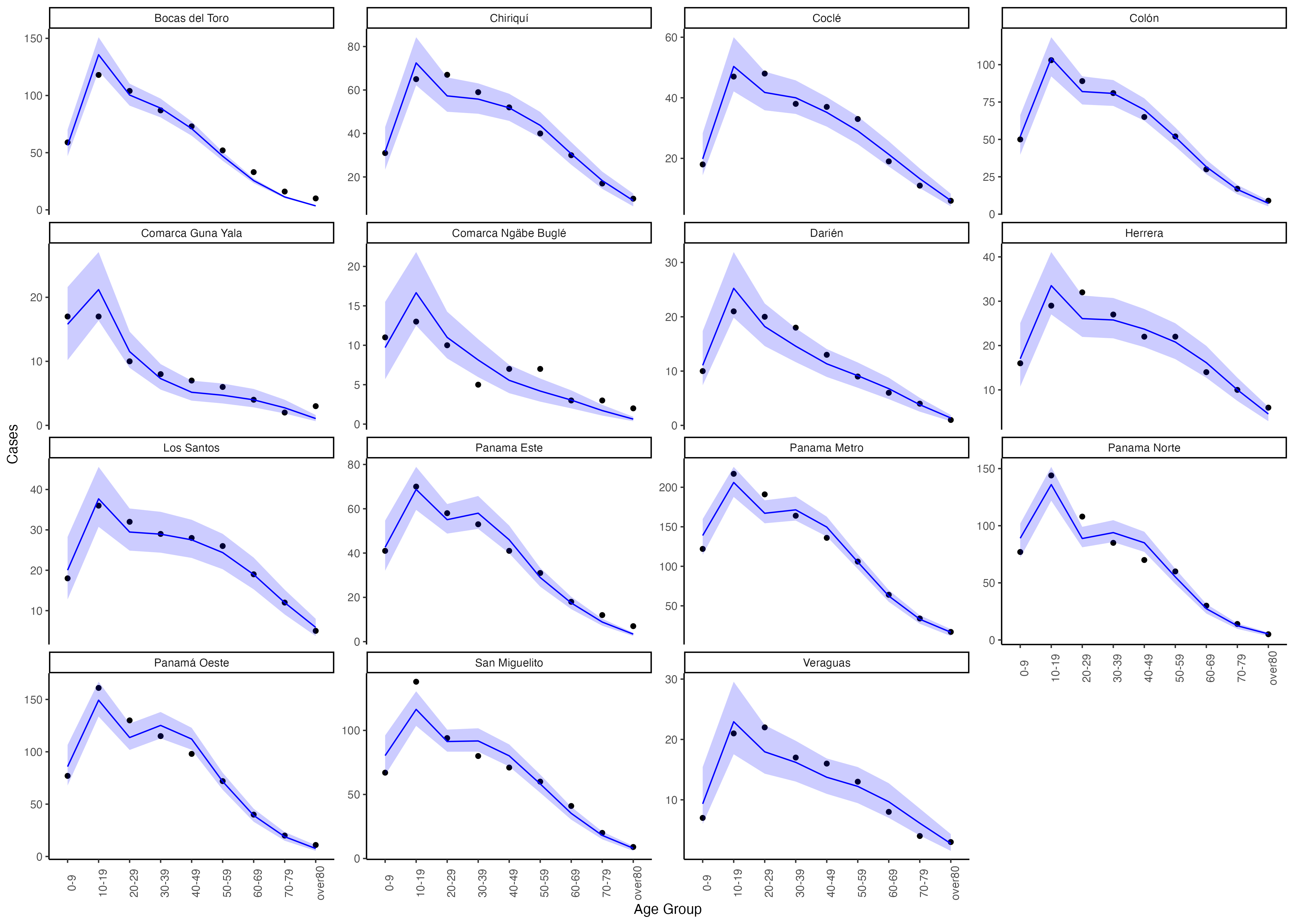


**Figure S4**. Fit of Model B, using a Poisson likelihood, calibrated to the age-distribution of the case-notification data reported at regional level in Panama. Each panel shows the observed case notification data by region and by age-group (black points) and the median of the model estimates (blue line) with the 95% CrI (shaded blue ribbon).


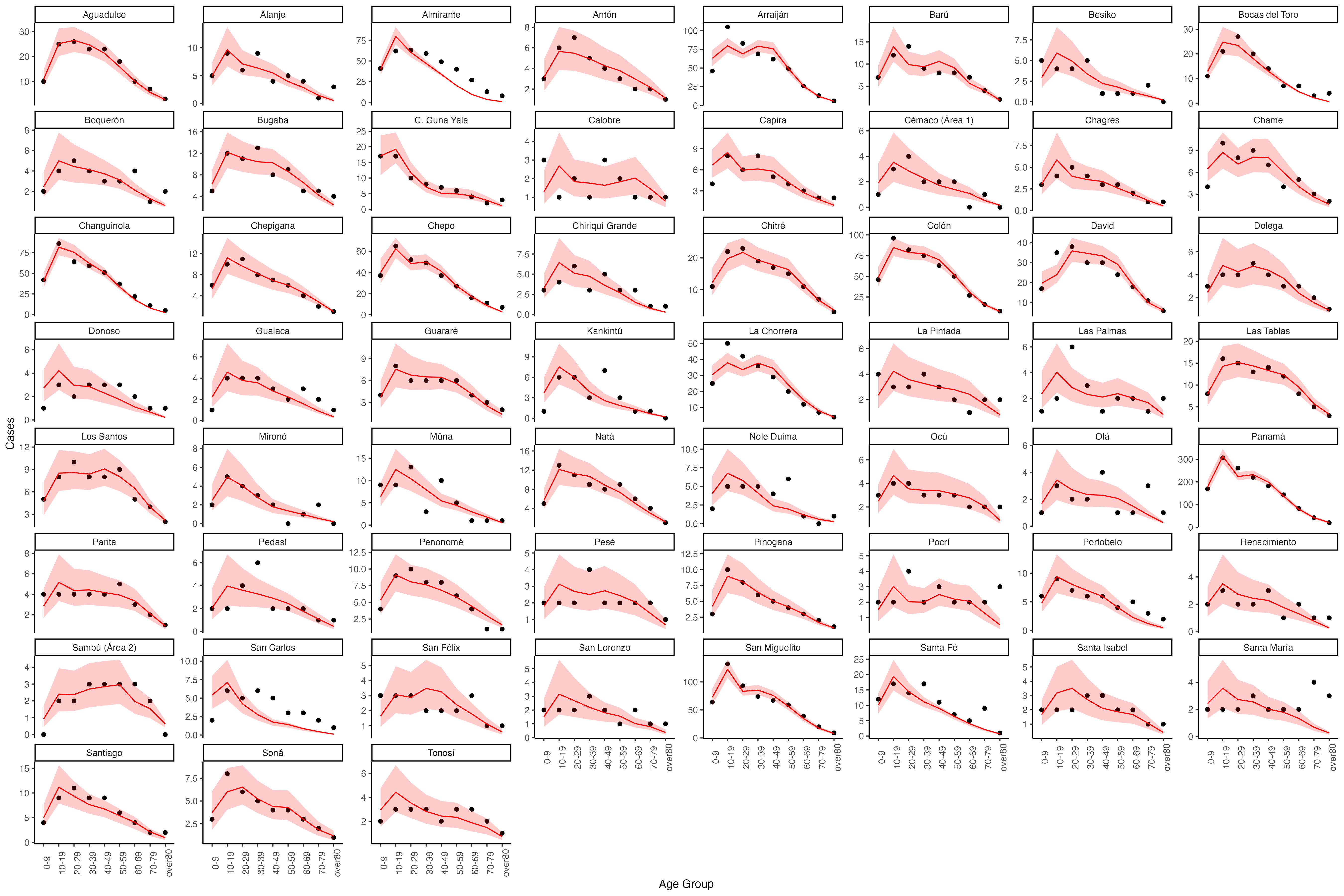


**Figure S5**. Fit of Model B calibrated to the age-distribution of the case-notification data reported at district-level in Panama. Each panel shows the observed case notification data by district by age-group (black points) and the median of the model estimates (red line) with the 95% CrI (shaded red ribbon).


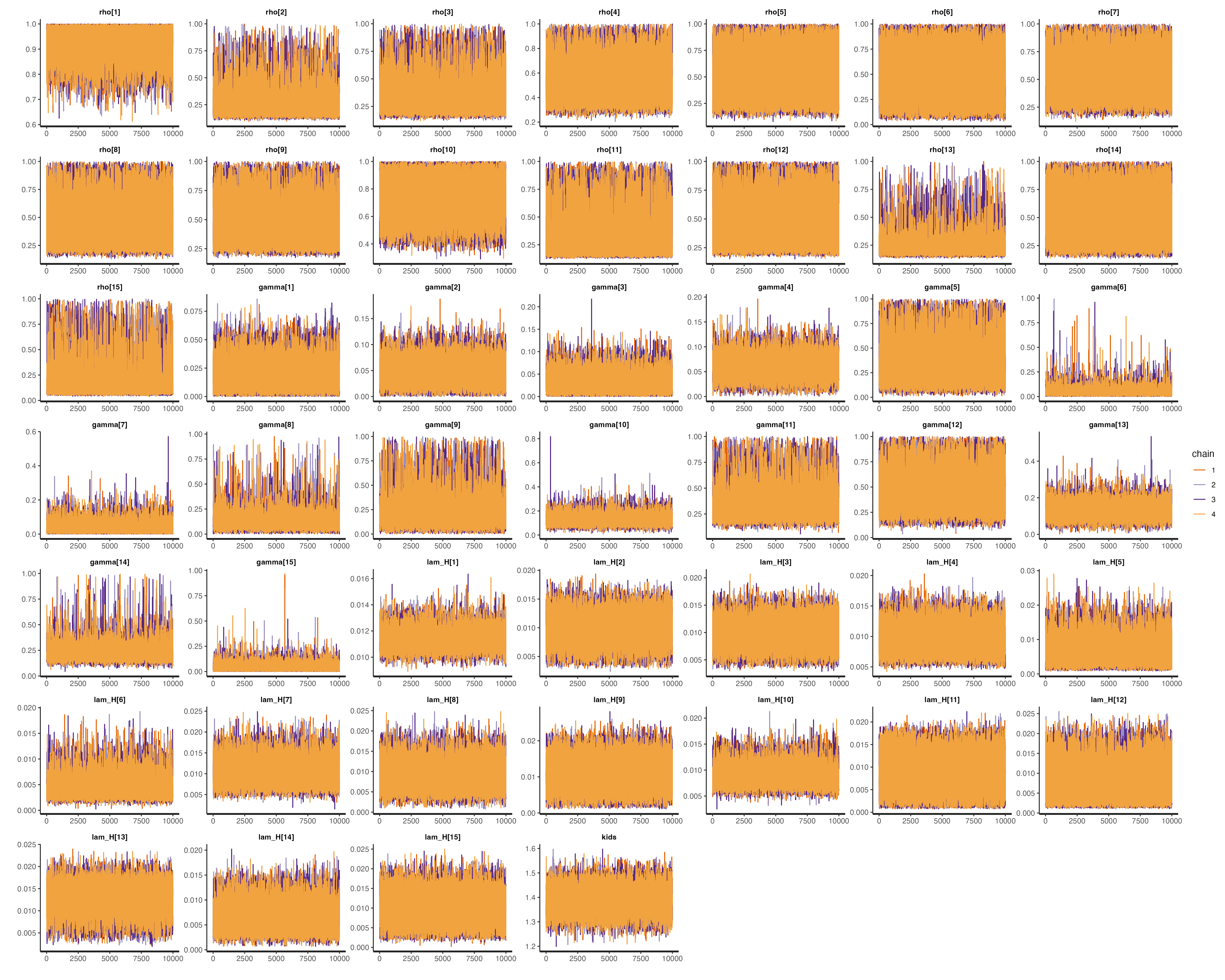


**Figure S6**. MCMC trace plots for all regional parameters in Model B, fitted using a Poisson likelihood. The plots show good chain mixing and no evidence of non-convergence across regions.


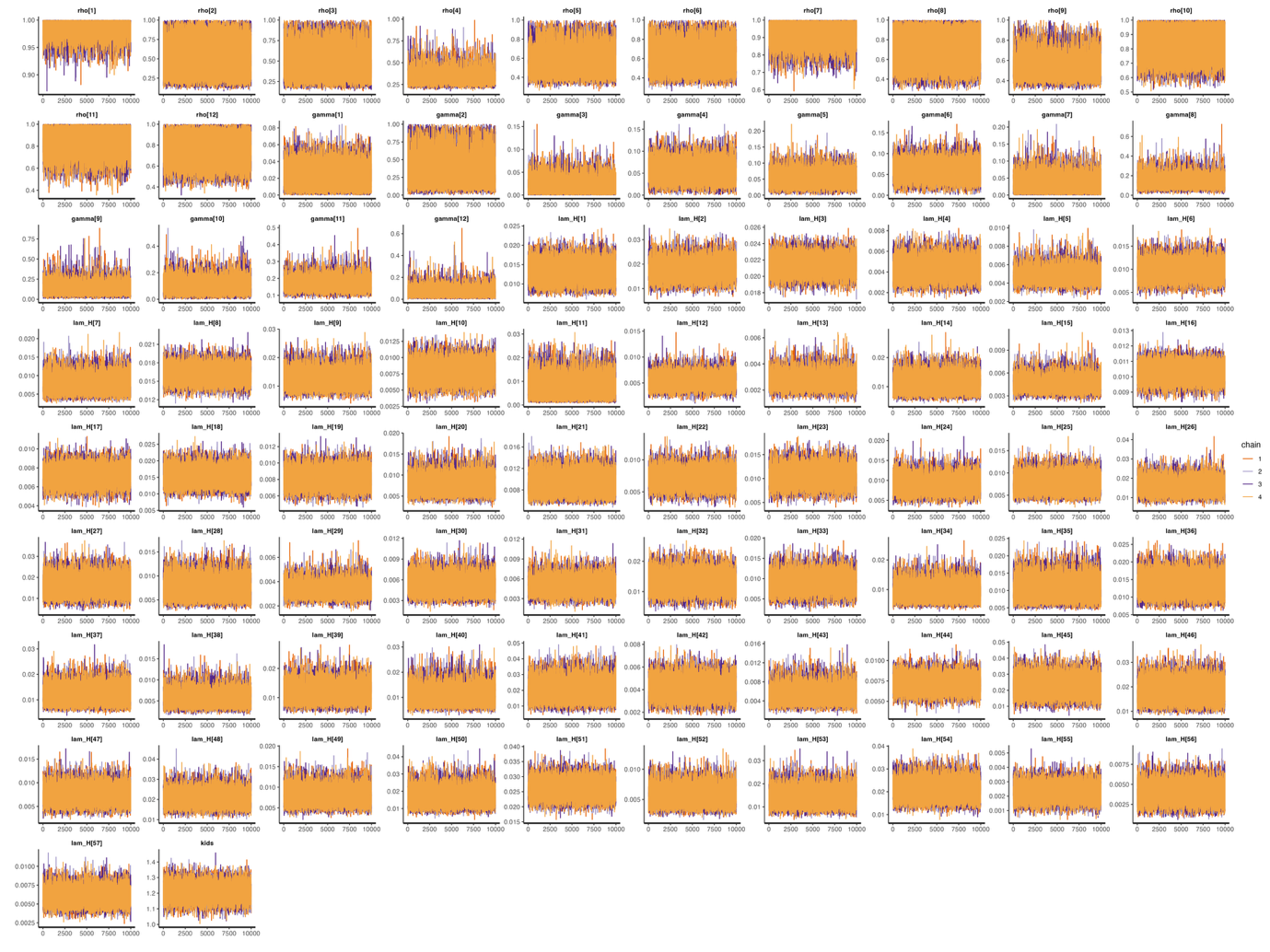


**Figure S7.** MCMC trace plots for all districts parameters in Model B, fitted using a Poisson likelihood. The plots show good chain mixing and no evidence of non-convergence across districts.

### Tables

**Table S1**. Incidence risk ratio (IRR) for reported dengue cases, hospitalisations and deaths by sex and age for two periods: before 2010 and from 2010 onward. For each exposure group, the table includes the IRR, 95% confidence Intervals (CI), standard error (std.error), and p.value using female (sex), 0-9 years (age) and the year 2000 as reference categories. The two periods correspond to different case-notification frameworks: before 2010, dengue was classified under the 1997 PAHO/WHO criteria with stricter confirmation requirements and centralized diagnostics; after 2010, the 2009 PAHO/WHO classification and decentralized rapid diagnostics expanded the clinical and laboratory criteria used for dengue reporting.

| Period | **2000-2009** | | | **2010-2024** | | |
| --- | --- | --- | --- | --- | --- | --- |
| Exposure | IRR | Std. | p.value | IRR | Std. | p.value |
| Dengue Cases |  | | |  | | |
| Female (baseline) | 1(1-1) | 0 | NA | 1(1-1) | 0 | NA |
| Male | 0.77 (0.75-0.79) | 0.01 | < 0.01 | 0.93 (0.92-0.94) | 0.01 | < 0.01 |
| age0-9 (baseline) | 1(1-1) | 0 | NA | 1(1-1) | 0 | NA |
| Age10-19 | 2.57 (2.44-2.71) | 0.03 | < 0.01 | 1.90 (1.86-1.94) | 0.01 | < 0.01 |
| Age20-29 | 2.91 (2.76-3.06) | 0.03 | < 0.01 | 1.70 (1.66-1.74) | 0.01 | < 0.01 |
| Age30-39 | 3.11 (2.95-3.27) | 0.03 | < 0.01 | 1.57 (1.53-1.60) | 0.01 | < 0.01 |
| Age40-49 | 3.33 (3.16-3.52) | 0.03 | < 0.01 | 1.46 (1.42-1.49) | 0.01 | < 0.01 |
| Age50-59 | 3.52 (3.32-3.73) | 0.03 | < 0.01 | 1.48 (1.44-1.52) | 0.01 | < 0.01 |
| Age60-69 | 3.15 (2.95-3.37) | 0.03 | < 0.01 | 1.35 (1.31-1.39) | 0.02 | < 0.01 |
| Age70-79 | 2.70 (2.48-2.93) | 0.04 | < 0.01 | 1.23 (1.18-1.28) | 0.02 | < 0.01 |
| Ageover80 | 3.89 (3.50-4.31) | 0.05 | < 0.01 | 1.01 (0.95-1.06) | 0.03 | < 0.01 |
| Hospitalisations |  | | |  | | |
| Female (baseline) | 1(1-1) | 0 | NA | 1(1-1) | 0 | NA |
| Male | 1.11 (1.08-1.13) | 0.02 | < 0.01 | 0.99 (0.97-1.01) | 0.01 | 0.51 |
| age0-9 (baseline) | 1(1-1) | 0 | NA | 1(1-1) | 0 | NA |
| Age10-19 | 0.65 (0.63-0.68) | 0.02 | < 0.01 | 0.83 (0.80-0.86) | 0.02 | < 0.01 |
| Age20-29 | 0.63 (0.60-0.66) | 0.01 | < 0.01 | 0.90 (0.87-0.93) | 0.02 | < 0.01 |
| Age30-39 | 0.66 (0.63-0.69) | 0.02 | < 0.01 | 1.02 (0.99-1.06) | 0.02 | 0.19 |
| Age40-49 | 0.65 (0.62-0.68) | 0.02 | < 0.01 | 1.09 (1.05-1.13) | 0.02 | < 0.01 |
| Age50-59 | 0.89 (0.85-0.93) | 0.02 | < 0.01 | 1.21 (1.16-1.25) | 0.02 | < 0.01 |
| Age60-69 | 1.11 (1.06-1.16) | 0.02 | < 0.01 | 1.13 (1.08-1.19) | 0.02 | < 0.01 |
| Age70-79 | - | - | - | 1.05 (0.97-1.13) | 0.04 | 0.22 |
| Ageover80 | - | - | - | 2.04 (1.93-2.16) | 0.03 | < 0.01 |
| Dengue deaths |  | | |  | | |
| Female (baseline) | 1(1-1) | 0 | NA | 1(1-1) | 0 | NA |
| Male | 0.24 (0.05-0.43) | 0.1 | < 0.01 | 0.99 (0.97-1.01) | 0.01 | 0.51 |
| age0-9 (baseline) | 1(1-1) | 0 | NA | 1(1-1) | 0 | NA |
| Age10-19 | -0.46 (-0.90--0.01) | 0.23 | < 0.01 | 0.83 (0.80-0.86) | 0.02 | < 0.01 |
| Age20-29 | -0.37 (-0.80-0.07) | 0.22 | 0.09 | 0.90 (0.87-0.93) | 0.02 | < 0.01 |
| Age30-39 | -0.40 (-0.84-0.05) | 0.23 | 0.08 | 1.02 (0.99-1.06) | 0.02 | 0.19 |
| Age40-49 | 0.19 (-0.21-0.61) | 0.21 | 0.37 | 1.09 (1.05-1.13) | 0.02 | < 0.01 |
| Age50-59 | 0.15 (-0.28-0.59) | 0.22 | 0.50 | 1.21 (1.16-1.25) | 0.02 | < 0.01 |
| Age60-69 | 0.48 (0.04-0.94) | 0.23 | < 0.01 | 1.13 (1.08-1.19) | 0.02 | < 0.01 |
| Age70-79 | 1.08 (0.63-1.54) | 0.23 | < 0.01 | 1.05 (0.97-1.13) | 0.04 | 0.22 |
| Ageover80 | 1.58 (1.12-2.05) | 0.23 | < 0.01 | 2.04 (1.93-2.16) | 0.03 | < 0.01 |

**Table S2**. AIC (Akaike Information Criterion) of each model (columns) applied to the respective delay distributions (rows) fitted by maximum likelihood estimation to the line list data in Panama for the time period 2005-2024.

| Category | weibull | gamma | lognormal | loglogistic | cauchy |
| --- | --- | --- | --- | --- | --- |
| National |  |  |  |  |  |
| Onset-to-Testing | 92225.4 | 90880.7 | 90062.0 | 91327.5 | 102375.9 |
| Onset-to-Reporting | 328044.8 | 324851.1 | 321891.5 | 325043.4 | 358400.9 |
| Onset-to-Hospitalisation | 28831.4 | 28512.2 | 28909.1 | 28751.4 | 30568.3 |
| Onset-to-Recovery | 4269.2 | 4269.5 | 4343.1 | 4305.4 | 4533.2 |
| Hospitalisation-to-Recovery | 3761.5 | 3701.3 | 3662.6 | 3680.0 | 4007.9 |
| Regional |  |  |  |  |  |
| Onset-to-Testing |  |  |  |  |  |
| Bocas del Toro | 11197.9 | 11103.4 | 11152.1 | 11296.5 | 12625.3 |
| Cocle | 3171.2 | 3134.3 | 3104.0 | 3146.1 | 3518.9 |
| Colon | 10560.2 | 10394.1 | 10164.4 | 10312.5 | 11589.2 |
| Chiriquí | 8838.5 | 8722.6 | 8704.4 | 8812.1 | 9820.1 |
| Darien | 1122.0 | 1099.2 | 1079.5 | 1101.9 | 1100.7 |
| Herrera | 3628.6 | 3565.3 | 3516.3 | 3565.4 | 4000.1 |
| Los Santos | 3114.0 | 3129.8 | 3212.3 | 3210.2 | 3339.2 |
| Panama Metro | 21019.8 | 20635.3 | 20350.8 | 20619.0 | 23119.5 |
| Panama Este | 5895.7 | 5813.3 | 5711.0 | 5814.7 | 6610.3 |
| Panama Oeste | 6319.2 | 6234.1 | 6251.4 | 6276.6 | 6822.7 |
| San Miguelito | 5844.1 | 5746.3 | 5706.7 | 5767.4 | 6384.2 |
| Veraguas | 1179.1 | 1138.4 | 1108.5 | 1120.1 | 1248.0 |
| Onset-to-Reporting |  |  |  |  |  |
| Bocas del Toro | 31192.0 | 30900.0 | 30568.9 | 30949.6 | 34459.2 |
| Cocle | 14122.5 | 14036.9 | 14030.7 | 14138.3 | 15451.2 |
| Colon | 32523.8 | 32308.9 | 32048.8 | 32425.8 | 35940.6 |
| Chiriquí | 29936.1 | 29733.3 | 29899.1 | 30028.2 | 32502.8 |
| Darien | 4929.2 | 4897.5 | 4815.8 | 4874.5 | 5408.6 |
| Herrera | 8009.4 | 7914.3 | 7853.2 | 7933.9 | 8783.6 |
| Los Santos | 11802.6 | 11721.4 | 11726.0 | 11855.0 | 13083.7 |
| Panama Metro | 73966.9 | 73241.1 | 72516.3 | 73201.0 | 80612.1 |
| Panama Este | 14793.7 | 14615.0 | 14348.2 | 14519.2 | 16173.8 |
| Panama Oeste | 35143.9 | 34673.8 | 34457.4 | 34668.5 | 37898.3 |
| San Miguelito | 34372.5 | 34025.4 | 33571.6 | 33875.0 | 37266.4 |
| Veraguas | 6136.5 | 6088.6 | 6136.0 | 6149.9 | 6652.0 |
| Onset-to-Hospitalisation |  |  |  |  |  |
| Bocas del Toro | 2251.6 | 2206.2 | 2216.5 | 2197.1 | 2319.3 |
| Cocle | 886.4 | 881.0 | 895.9 | 889.9 | 941.3 |
| Colon | 32523.8 | 32308.9 | 32048.8 | 32425.8 | 35940.6 |
| Chiriquí | 2644.2 | 2608.8 | 2610.3 | 2621.5 | 2844.3 |
| Darien | 368.7 | 367.9 | 372.1 | 375.7 | 414.3 |
| Herrera | 728.9 | 708.3 | 704.4 | 698.0 | 741.2 |
| Los Santos | 757.1 | 758.2 | 776.4 | 773.3 | 817.0 |
| Panama Metro | 7646.0 | 7574.1 | 7710.2 | 7651.2 | 8101.7 |
| Panama Este | 1501.9 | 1479.0 | 1498.1 | 1486.2 | 1585.8 |
| Panama Oeste | 4164.2 | 4143.8 | 4239.0 | 4182.7 | 4399.9 |
| San Miguelito | 3011.0 | 2982.7 | 3044.6 | 3000.1 | 3145.6 |
| Veraguas | 444.7 | 437.5 | 435.4 | 434.7 | 464.0 |
| Onset-to-Recovery |  |  |  |  |  |
| Bocas del Toro | 112.7 | 110.5 | 110.3 | 110.5 | 115.4 |
| Cocle | 70.6 | 71.0 | 72.1 | 72.1 | 74.5 |
| Colon | 267.4 | 264.9 | 268.6 | 262.8 | 272.5 |
| Chiriquí | 269.7 | 263.8 | 262.4 | 264.1 | 286.0 |
| Darien | 127.0 | 129.1 | 133.1 | 131.5 | 134.3 |
| Panama Metro | 1752.8 | 1749.3 | 1776.0 | 1762.6 | 1856.6 |
| Panama Este | 96.3 | 96.8 | 97.5 | 99.3 | 108.8 |
| Panama Oeste | 548.1 | 548.0 | 553.3 | 556.2 | 597.2 |
| San Miguelito | 916.6 | 921.3 | 941.9 | 928.6 | 963.2 |
| Hospitalisation-to-Recovery |  |  |  |  |  |
| Bocas del Toro | 85.2 | 84.8 | 85.1 | 86.6 | 98.1 |
| Cocle | 78.5 | 78.1 | 78.0 | 78.9 | 86.8 |
| Colon | 264.4 | 260.1 | 257.2 | 257.8 | 279.6 |
| Chiriqui | 204.0 | 201.0 | 192.6 | 195.0 | 219.9 |
| Darien | 98.2 | 96.3 | 96.1 | 97.0 | 103.4 |
| Panama Metro | 1616.3 | 1592.4 | 1578.4 | 1586.3 | 1724.3 |
| Panama Este | 113.6 | 112.9 | 111.8 | 113.2 | 125.5 |
| Panama Oeste | 435.7 | 428.3 | 422.0 | 421.1 | 448.5 |
| San Miguelito | 768.3 | 755.3 | 754.6 | 757.2 | 827.4 |
| Age |  |  |  |  |  |
| Paediatric (≤13) |  |  |  |  |  |
| Onset-to-Testing | 12839.0 | 12638.7 | 12487.1 | 12698.6 | 14367.4 |
| Onset-to-Reporting | 47538.6 | 47074.8 | 46443.6 | 47039.1 | 52427.7 |
| Onset-to-Hospitalisation | 4681.2 | 4599.1 | 4624.9 | 4611.8 | 4948.0 |
| Onset-to-Recovery | 358.1 | 350.7 | 343.1 | 345.4 | 380.0 |
| Hospitalisation-to-Recovery | 310.1 | 295.2 | 281.3 | 279.1 | 300.3 |
| Adult (≥14) |  |  |  |  |  |
| Onset-to-Testing | 64876.5 | 63949.9 | 63460.6 | 64275.9 | 71781.5 |
| Onset to-Reporting | 245307.8 | 242987.8 | 241117.7 | 243299.2 | 267560.2 |
| Onset-to-Hospitalisation | 21084.8 | 20883.3 | 21219.6 | 21070.6 | 22301.2 |
| Onset-to-Recovery | 245307.8 | 242987.8 | 241117.7 | 243299.2 | 267560.2 |
| Hospitalisation-to-Recovery | 3381.4 | 3328.3 | 3297.3 | 3312.3 | 3608.2 |
| Sex |  |  |  |  |  |
| Male |  |  |  |  |  |
| Onset-to-Testing | 42424.6 | 41749.4 | 41335.2 | 41873.8 | 46848.9 |
| Onset-to-Reporting | 151444.9 | 149864.1 | 148304.3 | 149743.2 | 165141.6 |
| Onset-to-Hospitalisation | 12878.7 | 12729.7 | 12901.3 | 12826.2 | 13637.0 |
| Onset-to-Recovery | 1898.9 | 1893.3 | 1919.1 | 1909.1 | 2010.3 |
| Hospitalisation-to-Recovery | 1687.0 | 1663.0 | 1641.3 | 1651.0 | 1795.9 |
| Female |  |  |  |  |  |
| Onset-to-Testing | 44901.2 | 15002.4 | 15237.2 | 15158.5 | 16116.5 |
| Onset-to-Reporting | 167439.7 | 44285.2 | 43923.9 | 44561.2 | 49992.3 |
| Onset-to-Hospitalisation | 15146.2 | 165913.9 | 164652.6 | 166243.1 | 183167.5 |
| Onset-to-Recovery | 2371.0 | 2378.8 | 2427.0 | 2398.8 | 2526.7 |
| Hospitalisation-to-Recovery | 2073.6 | 2037.9 | 2022.1 | 2030.3 | 2215.5 |

**Table S3**. Median and 95% Confidence Interval CI of the fitted delay distribution by category, calculated for the time period 2005-2024.

| Category | Delay | Median(days) | 95% CI |
| --- | --- | --- | --- |
| National | Onset-to-Testing | 3.48 | 3.39 - 3.57 |
|  | Onset-to-Reporting | 4.78 | 4.72 - 4.84 |
|  | Onset-to-Hospitalisation | 4.49 | 4.22 - 4.76 |
|  | Onset-to-Recovery | 7.82 | 6.47 - 8.85 |
|  | Hospitalisation-to-Recovery | 4.16 | 3.26 - 4.52 |
| Bocas del Toro | Onset-to-Testing | 3.85 | 3.76 - 3.95 |
|  | Onset-to-Reporting | 4.44 | 4.36 - 4.53 |
|  | Onset-to-Hospitalisation | 4.52 | 4.29 - 4.75 |
|  | Onset-to-Recovery | 7.38 | 5.91 - 8.85 |
|  | Hospitalisation-to-Recovery | 3.50 | 2.61 - 4.39 |
| Chiriquí | Onset-to-Testing | 3.48 | 3.39 - 3.57 |
|  | Onset-to-Reporting | 5.29 | 5.20 - 5.37 |
|  | Onset-to-Hospitalisation | 4.09 | 3.88 - 4.30 |
|  | Onset-to-Recovery | 7.82 | 6.88 - 8.77 |
|  | Hospitalisation-to-Recovery | 3.37 | 2.46 - 4.28 |
| Cocle | Onset-to-Testing | 3.92 | 3.71 - 4.14 |
|  | Onset-to-Reporting | 5.96 | 5.79 - 6.12 |
|  | Onset-to-Hospitalisation | 4.81 | 4.47 - 5.14 |
|  | Onset-to-Recovery | 8.25 | 5.96 - 10.54 |
|  | Hospitalisation-to-Recovery | 5.86 | 3.70 - 8.02 |
| Colon | Onset-to-Testing | 3.46 | 3.36 - 3.57 |
|  | Onset-to-Reporting | 5.31 | 5.21 - 5.42 |
|  | Onset-to-Hospitalisation | 4.09 | 3.86 - 4.31 |
|  | Onset-to-Recovery | 8.75 | 7.92 - 9.58 |
|  | Hospitalisation-to-Recovery | 4.62 | 3.79 - 5.45 |
| Darien | Onset-to-Reporting | 3.80 | 3.58-4.02 |
|  | Onset-to-Testing | 5.27 | 4.98 - 5.57 |
|  | Onset-to-Hospitalisation | 3.98 | 3.49 - 4.46 |
|  | Onset-to-Recovery | 5.92 | 4.92 - 6.93 |
|  | Hospitalisation-to-Recovery | 3.00 | 2.27 - 3.73 |
| Herrera | Onset-to-Testing | 3.37 | 3.21 - 3.53 |
|  | Onset-to-Reporting | 4.25 | 4.10 - 4.40 |
|  | Onset-to-Hospitalisation | 4.59 | 4.16 - 5.02 |
| Los Santos | Onset-to-Testing | 4.63 | 4.44 - 4.82 |
|  | Onset-to-Reporting | 4.61 | 4.48 - 4.74 |
|  | Onset-to-Hospitalisation | 4.46 | 4.10 - 4.83 |
|  | Onset-to-Recovery | 6.50 | 5.29 - 7.71 |
|  | Hospitalisation-to-Recovery | 2.92 | 2.29 - 3.55 |
| Panama Metro | Onset-to-Testing | 3.41 | 3.34 - 3.48 |
|  | Onset-to-Reporting | 4.78 | 4.72 - 4.84 |
|  | Onset-to-Hospitalisation | 4.40 | 4.30 - 4.51 |
|  | Onset-to-Recovery | 8.04 | 7.68 - 8.40 |
|  | Hospitalisation-to-Recovery | 4.42 | 4.10 - 4.74 |
| Panama Este | Onset-to-Testing | 3.32 | 3.19 - 3.45 |
|  | Onset-to-Reporting | 4.13 | 4.01 - 4.25 |
|  | Onset-to-Hospitalisation | 4.25 | 4.04 - 4.46 |
|  | Onset-to-Recovery | 7.74 | 6.47 - 9.00 |
|  | Hospitalisation-to-Recovery | 5.19 | 3.26 - 7.12 |
| Panama Norte | Onset-to-Testing | 3.04 | 2.95 - 3.14 |
|  | Onset-to-Reporting | 3.78 | 3.69 - 3.86 |
| Panama Oeste | Onset-to-Testing | 4.04 | 3.91 - 4.17 |
|  | Onset-to-Reporting | 4.47 | 4.39 - 4.54 |
|  | Onset-to-Hospitalisation | 4.65 | 4.51 - 4.78 |
|  | Onset-to-Recovery | 8.51 | 7.86 - 9.16 |
|  | Hospitalisation-to-Recovery | 4.90 | 4.10 - 5.70 |
| San Miguelito | Onset-to-Testing | 3.54 | 3.41 - 3.67 |
|  | Onset-to-Reporting | 4.84 | 4.74 - 4.93 |
|  | Onset-to-Hospitalisation | 4.62 | 4.47 - 4.78 |
|  | Onset-to-Recovery | 8.24 | 7.75 - 8.72 |
|  | Hospitalisation-to-Recovery | 4.16 | 3.80 - 4.52 |
| Veraguas | Onset-to-Testing | 4.50 | 4.17 - 4.84 |
|  | Onset-to-Reporting | 5.03 | 4.86 - 5.20 |
|  | Onset-to-Hospitalisation | 5.44 | 4.71 - 6.18 |
|  | Onset-to-Recovery | 6.83 | 2.88 - 10.79 |
|  | Hospitalisation-to-Recovery | 2.17 | 0.75 - 3.58 |
| Paediatric (≤ 13 years) | Onset-to-Testing | 3.28 | 3.20 - 3.36 |
|  | Onset-to-Reporting | 4.35 | 4.28 - 4.42 |
|  | Onset-to-Hospitalisation | 4.09 | 3.95 - 4.24 |
|  | Onset-to-Recovery | 3.56 | 2.96 - 4.16 |
|  | Hospitalisation-to-Recovery | 4.10 | 3.47 - 4.73 |
| Adult (≥14 years) | Onset-to-Testing | 3.63 | 3.59 - 3.67 |
|  | Onset-to-Reporting | 4.88 | 4.84 - 4.91 |
|  | Onset-to-Hospitalisation | 4.52 | 4.46 - 4.59 |
|  | Onset-to-Recovery | 8.14 | 7.90 - 8.38 |
|  | Hospitalisation-to-Recovery | 4.41 | 4.18 - 4.63 |
| Male | Onset-to-Testing | 4.62 | 4.58 - 4.66 |
|  | Onset-to-Reporting | 4.39 | 4.31 - 4.47 |
|  | Onset-to-Hospitalisation | 3.51 | 3.47 - 3.56 |
|  | Onset-to-Recovery | 8.05 | 7.68 - 8.41 |
|  | Hospitalisation-to-Recovery | 4.40 | 4.05 - 4.74 |
| Female | Onset-to-Testing | 4.84 | 4.80 - 4.88 |
|  | Onset-to-Reporting | 4.45 | 4.37 - 4.54 |
|  | Onset-to-Hospitalisation | 3.56 | 3.51 - 3.61 |
|  | Onset-to-Recovery | 8.05 | 7.76 - 8.35 |
|  | Hospitalisation-to-Recovery | 4.24 | 3.97 - 4.50 |

# **Table S4**. Median and 95% CI of the fitted delay distribution comparing two periods: before 2010 and from 2010. NA indicates insufficient data for analysis.

| Delay | Before 2010 | 2010 Onward |
| --- | --- | --- |
| Onset-to-Testing | NA | 3.1 (3.1-3.2) |
| Onset-to-Reporting | 6.1 (6.1-6.2) | 4.1 (4.0-4.1) |
| Onset-to-Hospitalisation | 4.4 (4.2-4.6) | 4.1 (4.1-4.2) |
| Onset-to-Recovery | 8.0 (7.7-8.3) | 8.2 (7.8-8.5) |
| Hospitalisation-to-Recovery | 4.3 (4.0-4.6) | 3.8 (3.5-4.1) |

# **Table S5**. Widely Applicable Information Criterion (WAIC)​ by Model version fitted to the province data.

| Version | Model A | Model B |
| --- | --- | --- |
| 1 | 19137.0 | 832.2 |
| 2 | 910.8 | 727.9 |

# **Table S6**. Parameters estimates (median and 95% CrI within brackets) obtained using Model B fitted to the age-stratified case data at the region-level in Panama. FOI indicates the Force of Infection (𝝀), indicates secondary infections and $\gamma$ indicates and relative reporting rate of primary vs secondary infections.

| Region | FOI _(95%CrI)_ | _(95%CrI)_ | $\gamma$_(95%CrI)_ |
| --- | --- | --- | --- |
| Bocas del Toro | 0.011 (0.010-0.013) | 0.946 (0.786-0.998) | 0.040 (0.006-0.145) |
| Coclé | 0.009 (0.005-0.015) | 0.297 (0.170-0.803) | 0.026 (0.002-0.079) |
| Darién | 0.010 (0.005-0.017) | 0.474 (0.225-0.944) | 0.033 (0.002-0.123) |
| Veraguas | 0.010 (0.003-0.018) | 0.125 (0.059-0.692) | 0.034 (0.002-0.135) |
| Comarca Ngäbe Buglé | 0.004 (0.002-0.010) | 0.409 (0.091-0.955) | 0.017 (0.001-0.046) |
| Chiriquí | 0.010 (0.004-0.015) | 0.218 (0.134-0.638) | 0.042 (0.005-0.095) |
| Colón | 0.009 (0.006-0.014) | 0.491 (0.299-0.919) | 0.057 (0.016-0.106) |
| Herrera | 0.008 (0.003-0.016) | 0.388 (0.190-0.931) | 0.118 (0.025-0.331) |
| Panama Este | 0.008 (0.006-0.013) | 0.749 (0.445-0.984) | 0.123 (0.057-0.229) |
| Panamá Oeste | 0.013 (0.005-0.019) | 0.214 (0.156-0.560) | 0.125 (0.050-0.223) |
| Los Santos | 0.010 (0.003-0.019) | 0.372 (0.224-0.908) | 0.178 (0.038-0.642) |
| San Miguelito | 0.005 (0.002-0.013) | 0.432 (0.183-0.948) | 0.219 (0.130-0.450) |
| C. Guna Yala | 0.006 (0.002-0.015) | 0.490 (0.177-0.959) | 0.251 (0.073-0.877) |
| Panama Metro | 0.009 (0.001-0.017) | 0.227 (0.145-0.886) | 0.298 (0.159-0.815) |
| Panama Norte | 0.006 (0.002-0.018) | 0.395 (0.207-0.941) | 0.398 (0.175-0.925) |

# **Table S7**. Parameter estimates (median and 95% CrI within brackets) obtained using Model B fitted to the age-stratified case data at the district-level in Panama. FOI indicates the Force of Infection (𝝀), indicates secondary infections and $\gamma$ indicates and relative reporting rate of primary vs secondary infections.

| Region | District | FOI | _(95%CrI)_ | $\gamma$_(95%CrI)_ |
| --- | --- | --- | --- | --- |
| Bocas del Toro | Almirante | 0.021 (0.019-0.023) | 0.842 (0.759-0.902) | 0.047 (0.012-0.099) |
| Bocas del Toro | Bocas del Toro | 0.016 (0.014-0.019) |  |  |
| Bocas del Toro | Changuinola | 0.010 (0.009-0.011) |  |  |
| Bocas del Toro | Chiriquí Grande | 0.008 (0.006-0.010) |  |  |
| Chiriquí | Alanje | 0.017 (0.011-0.025) | 0.475 (0.177-0.958) | 0.124 (0.033-0.216) |
| Chiriquí | Gualaca | 0.015 (0.009-0.023) |  |  |
| Chiriquí | Boquerón | 0.012 (0.008-0.018) |  |  |
| Chiriquí | David | 0.010 (0.007-0.014) |  |  |
| Chiriquí | Barú | 0.010 (0.006-0.013) |  |  |
| Chiriquí | Dolega | 0.009 (0.005-0.013) |  |  |
| Chiriquí | San Félix | 0.008 (0.005-0.012) |  |  |
| Chiriquí | Bugaba | 0.008 (0.005-0.011) |  |  |
| Chiriquí | Renacimiento | 0.007 (0.004-0.011) |  |  |
| Coclé | Natá | 0.013 (0.008-0.019) | 0.496 (0.477-0.500) | 0.021 (0.002-0.049) |
| Coclé | Aguadulce | 0.013 (0.008-0.018) |  |  |
| Coclé | Olá | 0.011 (0.007-0.018) |  |  |
| Coclé | La Pintada | 0.005 (0.003-0.008) |  |  |
| Coclé | Antón | 0.004 (0.003-0.006) |  |  |
| Coclé | Penonomé | 0.004 (0.003-0.006) |  |  |
| Colón | Portobelo | 0.017 (0.011-0.026) | 0.694 (0.402-0.981) | 0.128 (0.051-0.265) |
| Colón | San Lorenzo | 0.016 (0.009-0.027) |  |  |
| Colón | Chagres | 0.011 (0.007-0.017) |  |  |
| Colón | Colón | 0.009 (0.006-0.013) |  |  |
| Colón | Donoso | 0.007 (0.004-0.011) |  |  |
| Colón | Santa Isabel | 0.005 (0.003-0.009) |  |  |
| Comarca Guna Yala | Comarca Guna Yala | 0.006 (0.002-0.015) | 0.490 (0.177-0.959) | 0.251 (0.073-0.877) |
| Comarca Ngäbe Buglé | Nole Duima | 0.011 (0.007-0.019) | 0.532 (0.364-0.884) | 0.015 (0.001-0.054) |
| Comarca Ngäbe Buglé | Müna | 0.010 (0.006-0.016) |  |  |
| Comarca Ngäbe Buglé | Mironó | 0.009 (0.005-0.015) |  |  |
| Comarca Ngäbe Buglé | Besiko | 0.007 (0.004-0.012) |  |  |
| Comarca Ngäbe Buglé | Kankintú | 0.007 (0.004-0.011) |  |  |
| Darién | Santa Fé | 0.026 (0.021-0.031) | 0.515 (0.377-0.793) | 0.123 (0.035-0.291) |
| Darién | Pinogana | 0.007 (0.005-0.009) |  |  |
| Darién | Chepigana | 0.007 (0.005-0.009) |  |  |
| Darién | Cémaco (Área 1) | 0.007 (0.005-0.010) |  |  |
| Darién | Sambú (Área 2) | 0.006 (0.004-0.008) |  |  |
| Herrera | Santa María | 0.013 (0.008-0.021) | 0.876 (0.594-0.995) | 0.162 (0.110-0.246) |
| Herrera | Parita | 0.009 (0.006-0.017) |  |  |
| Herrera | Chitré | 0.007 (0.005-0.012) |  |  |
| Herrera | Ocú | 0.005 (0.003-0.009) |  |  |
| Herrera | Pesé | 0.005 (0.003-0.009) |  |  |
| Los Santos | Pocrí | 0.022 (0.012-0.034) | 0.753 (0.506-0.980) | 0.055 (0.011-0.156) |
| Los Santos | Pedasí | 0.018 (0.010-0.032) |  |  |
| Los Santos | Guararé | 0.015 (0.009-0.025) |  |  |
| Los Santos | Las Tablas | 0.012 (0.007-0.019) |  |  |
| Los Santos | Los Santos | 0.008 (0.005-0.013) |  |  |
| Los Santos | Tonosí | 0.004 (0.002-0.006) |  |  |
| Panama Este | Chepo | 0.015 (0.011-0.020) | 0.853 (0.645-0.992) | 0.090 (0.018-0.209) |
| Panama Metro | Panamá | 0.010 (0.003-0.015) | 0.268 (0.187-0.817) | 0.246 (0.157-0.433) |
| Panama Norte | Panamá | 0.010 (0.003-0.015) | 0.268 (0.187-0.817) | 0.246 (0.157-0.433) |
| Panamá Oeste | San Carlos | 0.020 (0.014-0.028) | 0.322 (0.234-0.521) | 0.051 (0.014-0.098) |
| Panamá Oeste | Arraiján | 0.005 (0.004-0.007) |  |  |
| Panamá Oeste | Chame | 0.004 (0.003-0.007) |  |  |
| Panamá Oeste | La Chorrera | 0.003 (0.002-0.005) |  |  |
| Panamá Oeste | Capira | 0.003 (0.002-0.004) |  |  |
| San Miguelito | San Miguelito | 0.005 (0.002-0.012) | 0.432 (0.183-0.948) | 0.219 (0.130-0.450) |
| Veraguas | Santiago | 0.020 (0.014-0.029) | 0.474 (0.401-0.499) | 0.026 (0.002-0.083) |
| Veraguas | Calobre | 0.005 (0.003-0.008) |  |  |
| Veraguas | Las Palmas | 0.005 (0.003-0.007) |  |  |
| Veraguas | Soná | 0.002 (0.001-0.003) |  |  |

**Table S8.** Spatial correlation diagnostic (Moran’s l) for FOI estimates.

| FOI | Moran's I | p.value | Standard deviate (z) |
| --- | --- | --- | --- |
| Region | -0.05 | 0.46 | 0.11 |
| District | 0.09 | 0.14 | 1.07 |

**Table S9**. Surveillance sensitivity (in %) by region, defined as the ratio of reported dengue cases to model-estimated infections, with 95% CrI intervals.

| Region | SS median (95% CrI) |
| --- | --- |
| Bocas del Toro | 2.50% (2.25%-2.72%) |
| Chiriquí | 0.64% (0.48%-1.23%) |
| Coclé | 0.80% (0.59%-1.41%) |
| Colón | 1.42% (1.05%-2.12%) |
| Guna Yala | 2.05% (0.94%-6.68%) |
| Ngäbe Buglé | 0.56% (0.24%-1.04%) |
| Darién | 1.22% (0.82%-1.94%) |
| Herrera | 1.36% (0.86%-2.93%) |
| Los Santos | 1.69% (1.18%-5.02%) |
| Panama Este | 2.54% (1.79%-3.39%) |
| Panama Metro | 1.18% (0.79%-5.91%) |
| Panama Norte | 2.48% (1.18%-7.86%) |
| Panamá Oeste | 0.90% (0.72%-1.82%) |
| San Miguelito | 1.70% (0.86%-3.73%) |
| Veraguas | 0.35% (0.24%-0.99%) |

**Table S10**. Reporting rate of secondary infections across two periods: before 2010 (ρ_1_), and from 2010 onward (ρ_2_). Estimates are presented as medians with 95% CrI, derived from Model version B2 at regional level in Panama.

| **Region** | ρ_1_ **_(95%CrI)_** | ρ_2_ **_(95%CrI)_** |
| --- | --- | --- |
| **Bocas del Toro** | 0.534 (0.179-0.969) | 0.957 (0.828-0.998) |
| **Chiriquí** | 0.448 (0.220-0.933) | 0.155 (0.105-0.391) |
| **Coclé** | 0.256 (0.130-0.805) | 0.253 (0.166-0.586) |
| **Colón** | 0.453 (0.177-0.954) | 0.429 (0.306-0.802) |
| **Guna Yala** | 0.136 (0.032-0.869) | 0.493 (0.190-0.960) |
| **Ngäbe Buglé** | 0.382 (0.094-0.946) | 0.383 (0.082-0.947) |
| **Darién** | 0.440 (0.133-0.952) | 0.428 (0.217-0.924) |
| **Herrera** | 0.274 (0.119-0.899) | 0.347 (0.218-0.866) |
| **Los Santos** | 0.184 (0.104-0.846) | 0.356 (0.253-0.855) |
| **Panamá Este** | 0.991 (0.954-1.000) | 0.777 (0.488-0.987) |
| **Panama Metro** | 0.117 (0.096-0.158) | 0.141 (0.123-0.194) |
| **Panama Norte** | 0.522 (0.236-0.959) | 0.388 (0.205-0.938) |
| **Panamá Oeste** | 0.232 (0.029-0.907) | 0.231 (0.142-0.871) |
| **San Miguelito** | 0.034 (0.001-0.821) | 0.316 (0.160-0.905) |
| **Veraguas** | 0.534 (0.179-0.969) | 0.087 (0.055-0.279) |
